# Optogenetic restoration of high sensitivity vision with bReaChES, a red-shifted channelrhodopsin

**DOI:** 10.1038/s41598-022-23572-4

**Published:** 2022-11-11

**Authors:** Lay Khoon Too, Weiyong Shen, Dario A. Protti, Atomu Sawatari, Dylan A. Black, Catherine A. Leamey, Jin Y. Huang, So-Ra Lee, Ashish E. Mathai, Leszek Lisowski, John Y. Lin, Mark C. Gillies, Matthew P. Simunovic

**Affiliations:** 1 Save Sight Institute, The University of Sydney, Sydney Eye Hospital South Block, 8 Macquarie St, Sydney, NSW 2000 Australia; 2 Sydney Eye Hospital, 8 Macquarie St, Sydney, NSW 2000 Australia; 3 Neuroscience, School of Medical Sciences, Faculty of Medicine and Health, The University of Sydney, Sydney, NSW 2006 Australia; 4 Education Innovation Theme, School of Medical Sciences, Faculty of Medicine and Health, The University of Sydney, Sydney, NSW 2006 Australia; 5 Children’s Medical Research Institute, The University of Sydney, Sydney, NSW 2006 Australia; 6 Laboratory of Molecular Oncology and Innovative Therapies, Military Institute of Medicine, 04-141 Warsaw, Poland; 7 School of Medicine, College of Health and Medicine, University of Tasmania, Tasmania, 7000 Australia

**Keywords:** Eye diseases, Retinal diseases, Vision disorders, Preclinical research, Genetics research

## Abstract

The common final pathway to blindness in many forms of retinal degeneration is the death of the light-sensitive primary retinal neurons. However, the normally light-insensitive second- and third-order neurons persist optogenetic gene therapy aims to restore sight by rendering such neurons light-sensitive. Here, we investigate whether bReaChES, a newly described high sensitivity Type I opsin with peak sensitivity to long-wavelength visible light, can restore vision in a murine model of severe early-onset retinal degeneration. Intravitreal injection of an adeno-associated viral vector carrying the sequence for bReaChES downstream of the calcium calmodulin kinase IIα promoter resulted in sustained retinal expression of bReaChES. Retinal ganglion cells (RGCs) expressing bReaChES generated action potentials at light levels consistent with bright indoor lighting (from 13.6 log photons cm^−2^ s^−1^). They could also detect flicker at up to 50 Hz, which approaches the upper temporal limit of human photopic vision. Topological response maps of bReaChES-expressing RGCs suggest that optogenetically activated RGCs may demonstrate similar topographical responses to RGCs stimulated by photoreceptor activation. Furthermore, treated dystrophic mice displayed restored cortical neuronal activity in response to light and rescued behavioral responses to a looming stimulus that simulated an aerial predator. Finally, human surgical retinal explants exposed to the bReaChES treatment vector demonstrated transduction. Together, these findings suggest that intravitreal gene therapy to deliver bReaChES to the retina may restore vision in human retinal degeneration in vivo at ecologically relevant light levels with spectral and temporal response characteristics approaching those of normal human photopic vision.

## Introduction

Inherited retinal degeneration (IRD)—which afflicts 1 in 3000—is now the most common cause of blindness in people of working age^1^. Although specific gene therapy for one form of IRD caused by homozygous loss of function mutations to the gene encoding RPE65, has now received regulatory approval, it is only suitable for perhaps 1 in 300,000–1.2 million^2,3^. This fact highlights one of the problems in developing gene-specific therapy for IRD, i.e. the significant underlying genetic diversity: at present, more than 300 genes or loci have been implicated in the etiology of IRD^4^. This diversity is compounded by the fact that around 30% of patients seen in specialist clinics have no causative mutation identified, even after extensive genetic investigation^5^. A further problem is that causative gene-specific gene therapy approaches may be inappropriate for patients with end-stage disease in whom there is irreversible loss of cellular structures required to support the “rescue” of vision. Although patients with so-called "end-stage" disease were the target group for electronic retinal prostheses, no device previously approved by regulatory authorities^6,7^ is currently available commercially, primarily due to low uptake. Therefore, there is a significant and presently unmet need for causative gene independent approaches to restoring vision in IRD: optogenetics is one such approach^8^, which has recently translated to Phase I studies^9^. Optogenetics has also been proposed as a possible strategy for restoring central vision in patients with advanced atrophic age-related macular degeneration (aARMD), a leading cause of blindness in those of retirement age^10^. However, given the inherent difficulties of precisely targeting therapy to the macula, the success of optogenetic vision restoration in aARMD will be crucially dependent on its interaction with photoreceptor-mediated vision in the well-preserved perimacular region and retinal periphery. At present, there are limited data on the effects of optogenetic gene expression in normal retina.

The final common pathway to vision loss in IRD and aARMD is the loss of the light-sensitive primary retinal neurons: the rods and cones. The majority of optogenetic approaches achieve vision restoration through the expression of either Type I (microbial)^10–12^ or Type II (animal) opsins^13–15^ in the ordinarily light-insensitive secondary (e.g. bipolar cells) or tertiary (e.g. retinal ganglion cells (RGCs)) neurons, which persist even in advanced IRD and aARMD. Type I opsins offer the advantage of simplicity: these molecules both detect light and act as the means of permitting changes in membrane potential, which in turn signal light detection^16^. Once activated through the photoisomerization of all-trans-retinal to 13-cis retinal, they return to their light-sensitive state through a process of molecular relaxation (as opposed to a complex cell-mediated process)^8^. Type II opsins, by contrast, signal the absorption of light at a cellular level via a complex signal transduction cascade. This cascade offers inherent signal amplification and, therefore, greater sensitivity^8^. A further advantage is that human Type II opsins may prove to be better tolerated by the human immune system. However, they suffer from the inherent limitation of being dependent on signal transduction cascades which are comparatively slow in their kinetics. This is compounded by their dependence on cell-mediated processes to return to their light-sensitive state. These processes are profoundly impaired in IRD and aARMD^8^.

Here, we describe the use of a newly described Type I opsin, bReaChES^17,18^, to restore vision in a murine model of severe, early-onset retinal degeneration. We have previously reviewed optogenetic approaches to vision restoration^8^ and have outlined the features of ideal, ecologically relevant, optogenetic molecules^8^. We selected bReaChES as a suitable candidate optogenetic molecule for vision restoration for two reasons. First, its peak spectral sensitivity is broad, lying at about 570–590 nm (yellow-green/yellow)^18^, which is close to the peak sensitivity of human vision under daylight (photopic) conditions^19^. This makes it well-suited to both the spectral transmission window of the human eye and to the emission spectrum of commonly encountered environmental light sources^8^. Second, it possesses high channel conductance, thereby making it theoretically superior to other previously described opsins—including its parent molecule ReaChR^11,12^—in terms of achievable sensitivity for equivalent protein expression. Therefore, we reasoned that bReaChES might provide vision restoration with greater light sensitivity than previously employed Type I opsins and consistent with environmental illumination levels. Our study demonstrates that bReaChES restores vision in a murine model of severe early-onset IRD under ambient lighting conditions, consistent with the predicted > 1 log unit improvement (from recently published in silico modelling) over other microbial opsins, such as ChrimsonR^20^. This high sensitivity does not come at the expense of poor temporal resolution: treated retinal ganglion cells could track 50 Hz flicker, which approaches the temporal limits of human vision. Furthermore, bReaChES expressed under the control of the calmodulin kinase IIα (*CamkIIα*) promoter – which is upregulated in IRD^21^—demonstrates long-term transduction and restores light-activated cFos expression in the visual cortex and appropriate behavioral responses to stimuli simulating a swooping predator.

## RESULTS

### Intravitreal injection of an AAV vector with CamkIIα promoter drives strong expression of bReaChES in the degenerate retina

**Figure 1:**
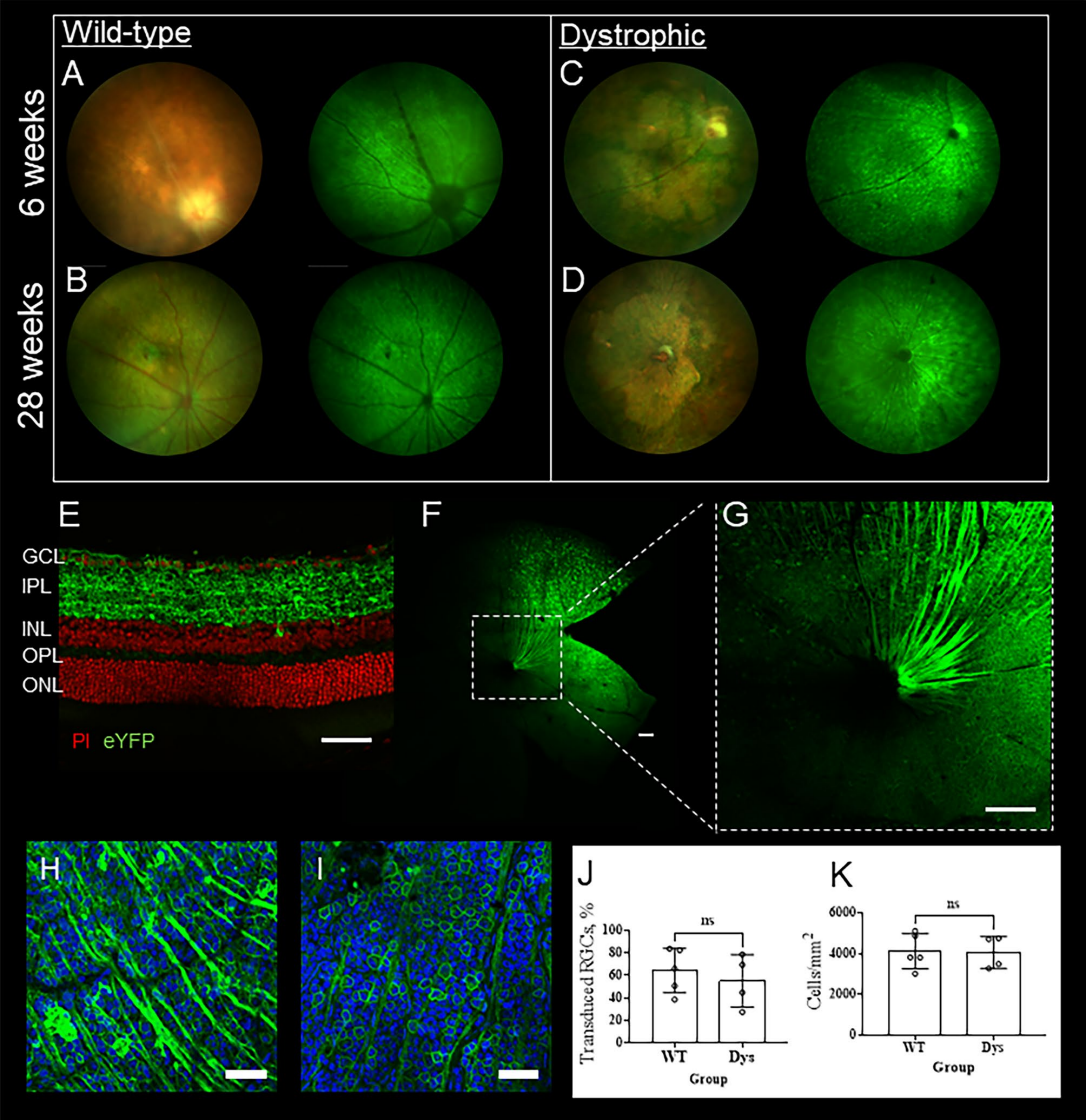
Prolonged expression of bReaChES in the retinal ganglion cells of wild-type (WT) and dystrophic (Dys) mouse retinae. (**A**–**D**) Representative fundus images (left: color, right: short-wavelength fluorescent fundus photography) of WT and dystrophic mice 6 (**A**,**C**) and 28 (**B**,**D**) weeks post-treatment. Green fluorescence indicates bReaChES-eYFP expression. (**E**–**H**) Representative WT retinal section (E) and flatmount (**F**–**G**) images that show membrane expression of bReaChES in GCL, IPL and nerve fibers. Representative retinal flatmounts from WT (H) and dystrophic (**I**) mouse retinae that show membrane expression of bReaChES in individual retinal ganglion cells. (**J**) The transduction rate of optogenetic vector in RGL is not significantly different between WT and Dys mice 6 months post injection (N = 5 WT and N = 4 Dys). (**K**) The density of RGL measured is not significantly different between WT and Dys mice 6 months post injection (N = 5 WT and N = 4 Dys). Error bars indicate ± SD. White scale bar = 50um. (Abbreviations: eYFP = enhanced yellow fluorescence protein; PI = propidium iodide; GCL = ganglion cell layer; IPL = inner plexiform layer; INL = inner nuclear layer; OPL = outer plexiform layer; ONL = outer nuclear layer).

Our purpose-bred iCre-DTA176 mouse, which expresses an attenuated diphtheria toxin under the control of the rhodopsin promoter, acts as an animal model of severe and early-onset retinal degeneration^22^. We assessed retinal transduction in vivo at 6 and 28 weeks following intravitreal injection of an AAV2 viral vector (*rAAV2/CamkIIα-bReaChES-TS-eYFP*) in these mice. This vector included the *eYFP* reporter gene to permit evaluation of expression in vivo using fundus fluorescent photography. Fundus fluorescent imaging confirmed the widespread expression of the viral vector visible in RGCs 6 weeks post-injection, which was sustained through to 28 weeks (Fig. 1A–D). Microscopic examination of retinal sections and flatmounts collected from mice 12 months after injection demonstrates widespread and robust membrane expression of bReaChES, primarily in the retinal ganglion cell layer and inner retina (Fig. 1E–I). The transduction rate of RGCs was up to 84% and 79% in wild-type (WT) and dystrophic retinae, respectively, at 6-months post-injection (Fig. 1H–J). This rate was not significantly different between WT (64% ± 20%) and dystrophic (55% ± 23%) mice (Fig. 1H–J, *p* = 0.54, unpaired *t*-test); nor did the two groups differ in their ganglion cell density (Fig. 1I,K, *p* = 0.90, unpaired *t*-test). Concomitantly, spectral domain-optical coherence tomographic imaging (SD-OCT) was performed to examine retinal structure 3 months after injection. As shown in Fig. S1, there were no apparent structural abnormalities in the retinae of bReaChES-treated dystrophic and WT mice due to treatment. The mean retinal thicknesses of the bReaChES-treated WT and dystrophic mice were 228.6um ± 11.5um (mean ± SD) and 98.9um ± 12.5um, respectively, which are not significantly different from their untreated counterparts (WT untreated: 235.9um ± 4.9um, *p* = 0.19, Welch's *t*-test; Dystrophic untreated: 104.7um ± 3.6um, p = 0.47, unpaired *t*-test). Differences between both treated and untreated dystrophic animals and their wild-type counterparts were significantly different (P < 0.001%). These observations suggest bReaChES optogenetic treatment targeting RGCs/inner retina does not cause structural damage.

### bReaChES expression in retinal ganglion cells mediates novel intrinsic light sensitivity

**Figure 2:**
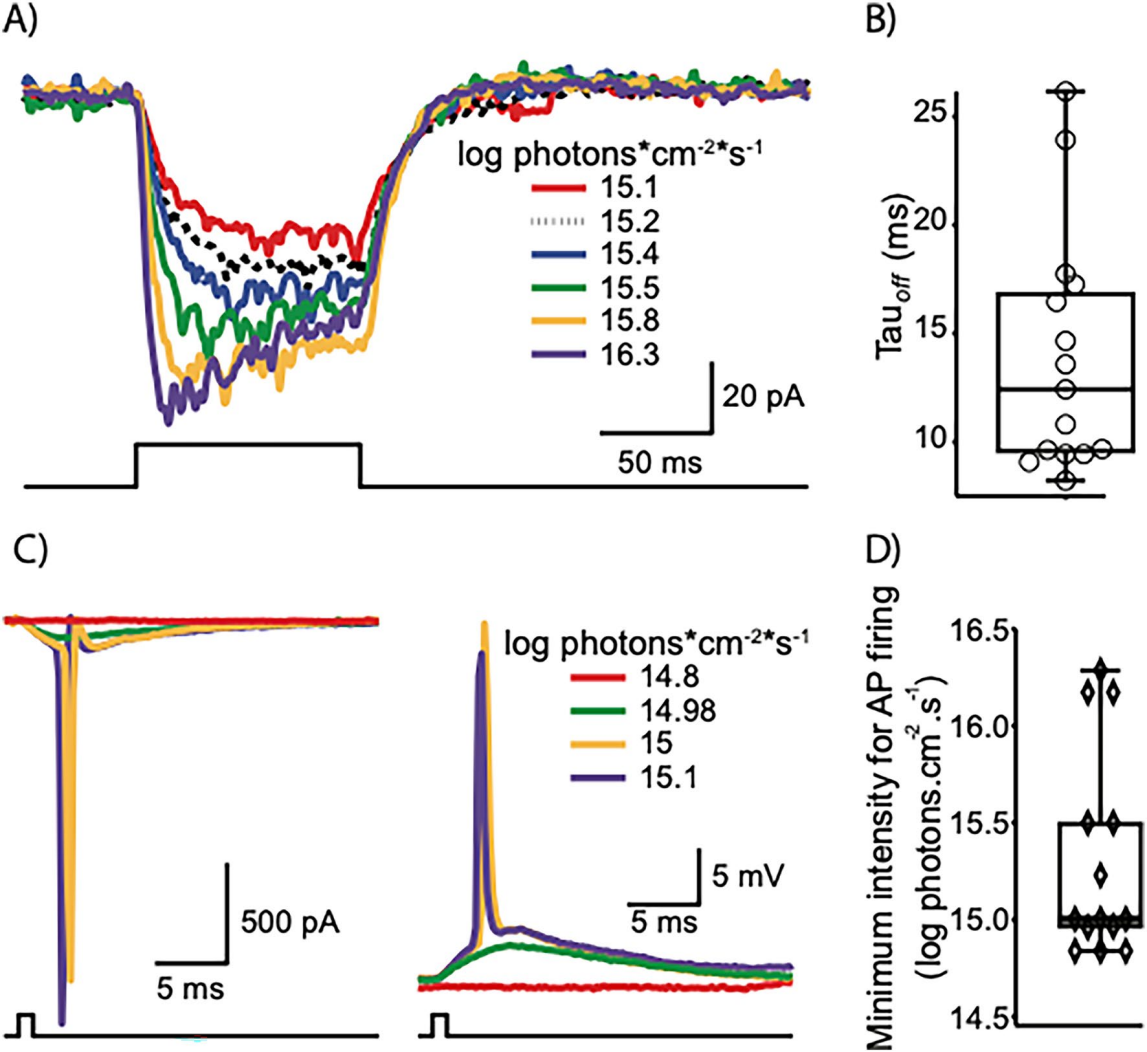
bReaChES expression in dystrophic mouse retina confers novel light sensitivity to RGCs. (**A**) Representative photocurrents recorded from a retinal ganglion cell in response to a 100 ms light pulse at different intensities (15.1 to 16.3 log photons cm^2^ s^−1^); a monoexponential function was fit to the tail currents at the end of the light pulse to calculate the off kinetics (τ_off_). (**B**) Distribution of τ_off_ quantified in retinal ganglion cells (n = 16) (**C**) Photocurrent (left) and voltage (right) traces elicited by 1 ms pulses of different intensity (14.8 to 15.1 log photons cm^2^ s^−1^). Intensities above 14.98 photons cm^2^ s^−1^generated photocurrents that activated voltage-gated sodium channels, as evidenced by the large, transient inward currents. The amplitude of the voltage response increased with increasing intensities. In this cell, intensities equal to (and above) 15 log photons cm^2^ s^−1^ elicited action potentials. (**D**) Threshold intensity required to elicit an action potential for a 1 ms light pulse (n = 14 cells). All responses were elicited by full-field illumination with λ = 565 nm following bleaching by intense white light (see methods).

We next asked whether the appropriate membrane expression of bReaChES in RGCs of treated animals could drive intrinsic photosensitivity. Furthermore, we sought to elucidate the temporal and spatial response characteristics of such sensitized cells. Single-cell patch-clamp recordings were obtained from RGCs expressing bReaChES in dystrophic mice unless otherwise stated. To established whether intrinsic properties of RGCs were altered in the DTA model or by viral expression of bReaChES, we measured their input resistance, spike height and spike width. Input resistance of bReaChES-expressing RGCs from dystrophic mice was 303 ± 65 MΩ (n = 10 cells, range: 66–633 MΩ), similar to that of RGCs from wild-type animals 360 ± 49 MΩ (n = 6 cells, range: 111–501 MΩ) (p > 0.5). No significant differences were observed in spike height (71 ± 12 mV in DTA vs 70 ± 2 mV) and width (846 ± 90 µsec vs 820 ± 134 µsec) between RGCs from bReaChes-expressing dystrophic retinae and control wild-type retinae (p > 0.7 and p > 0.8 respectively). Stimulation with 100 ms square light pulses (full field stimulation, λ = 565 nm) whilst holding RGCs at −60 mV in voltage-clamp mode induced inward currents of increasing magnitude as light intensity was increased (Fig. 2A). Time to peak amplitude decreased with increasing light intensity and 16.3 log photons cm^−2^ s^−1^ (the maximum light intensity tested) induced currents that rapidly reached peak amplitude and then decayed to a lower magnitude under constant illumination. The mean time constant of channel closure (τ_off_) was 14.1 ± 1.2 ms (n = 16 cells, Fig. 2B) as estimated from fitting a monoexponential function to the tail currents at the end of the light pulse.

We next aimed to determine the minimum light intensity required for a 1 ms full-field flash of 565 nm to elicit an action potential. Figure 2C shows responses to brief (1 ms) full-field stimulation in voltage- and current-clamp mode at sub- and supra-threshold intensities for the same cell. In this cell, flash intensities below 14.9 log photons cm^−2^ s^−1^ did not produce any responses whilst intensities above 14.93 log photons cm^−2^ s^−1^ (not shown) elicited inward currents whose magnitude increased with intensity. Moreover, flash intensities of 15 log photons cm^−2^ s^−1^ led to the generation of action currents due to escape of voltage control of unclamped action potentials (Fig. 2C, left traces). Recordings from the same cell in current clamp mode showed no response for the lowest intensity tested (14.8 log photons cm^−2^ s^−1^). Flashes of 14.98 log photons cm^−2^ s^−1^ intensity elicited short-latency depolarizing responses with a typical time course of an excitatory postsynaptic potential and further increments in light intensity resulted in changes in membrane potential of larger amplitude and a single action potential (Fig. 2C, left traces). The mean minimum intensity required to elicit an action potential with a 1 ms full-field flash was 15.3 1 ± 0.1 log photons.cm^−2^ s^−1^ (Fig. 2D, n = 14 cells; median = 15 log photons cm^−2^ s^−1^) at a mean latency of 2.3 ± 0.5 ms (n = 14 cells).

**Figure 3:**
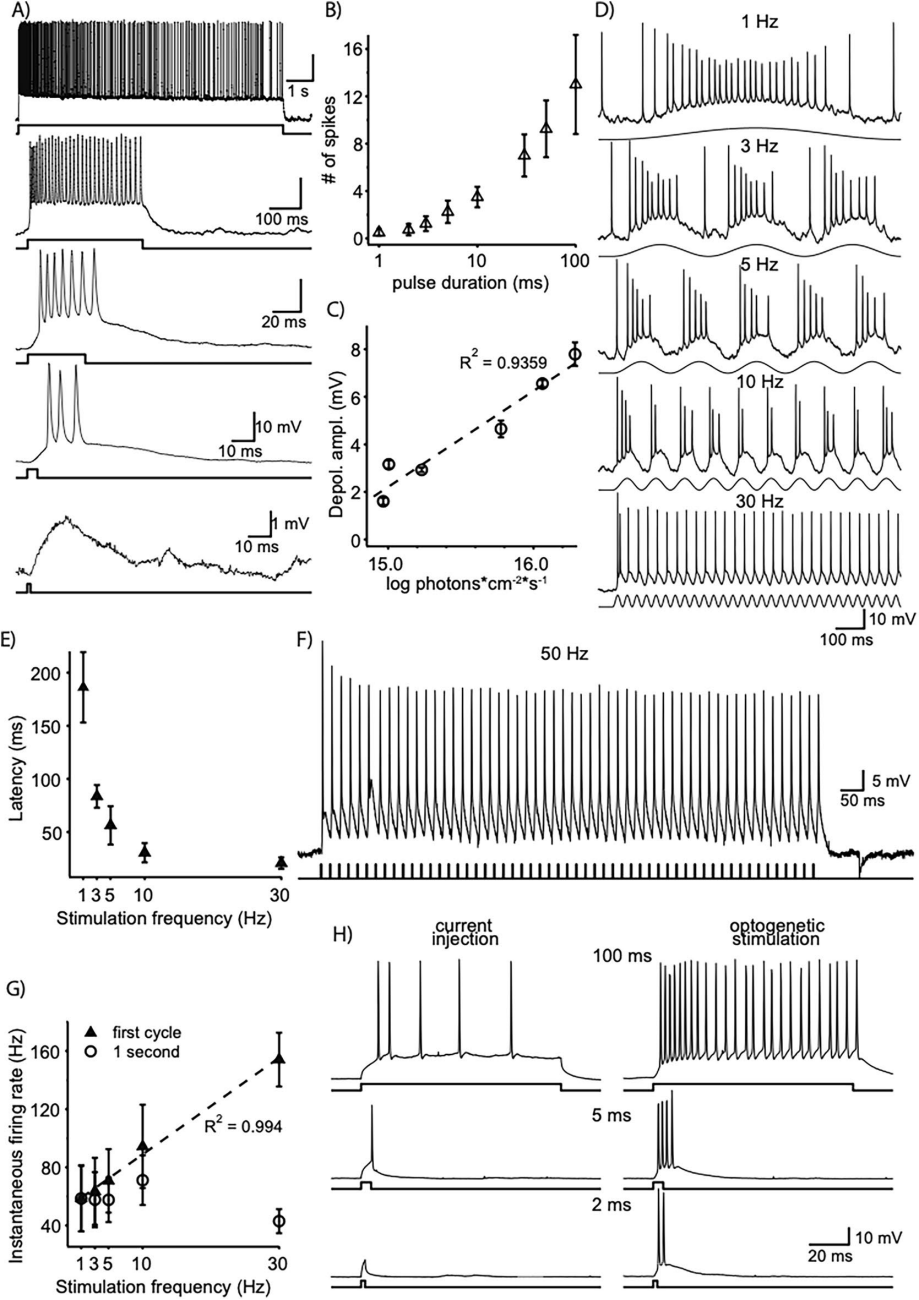
bReaChES expression confers high sensitivity to light. (**A**) Membrane potential recordings in response to light flashes of increasing durations (bottom to top: 1.5, 4.5, 40, 365 ms and 9.9 s) and constant intensity (14.4 log photons cm^−2^ s^−1^). A 1.5 ms flash produced a subthreshold response (bottom trace). Increase in flash duration elicited action potentials and increased the number of action potentials. (**B**) Duration-response function of the bReaChES-induced spike response. Number of spikes increased with duration. Stimulus intensity = 15.5 log photons cm^−2^ s^−1^. (**C**) Intensity-response function of the magnitude of the bReaChES-induced EPSP-like depolarization (action potentials were filtered out). A 1 ms flash was delivered at 6 different intensities (14.9 to 16.3 log photons cm^−2^ s^−1^). Symbols indicate mean ± SEM (n = 6 cells). (**D**) Membrane potential responses to sinusoidal modulation of light at 1, 3, 5, 10 and 30 Hz (top to bottom) in a RGC of a bReaChES-expressing dystrophic mouse. Sinusoidal stimulation resulted in reliable action potential firing for frequencies up to 30 Hz. Peak intensity of sinusoidal inputs = 14.24 log photons cm^−2^ s^−1^. (**E**) Latency to first spike elicited by 5 sinusoidal frequencies (n = 4 cells). (**F**) Short duration (1 ms, 16.9 log photons cm^−2^ s^−1^, λ = 470 nm) square pulses delivered to a 27.5 µm × 27.5 µm patch area over the cell soma reliably elicited action potentials in an RGC of bReaChES-expressing dystrophic mouse. (**G**) Instantaneous firing rate for the first cycle at each frequency (filled triangles) and for the first second of stimulation (empty circles). (**H**) Comparison of membrane potential responses to 3 current injection (left, 150 pA) and light (right, 15.1 log photons cm^−2^ s^−1^) pulses for 3 different durations (bottom to top: 2, 5 and 100 ms). Responses (except in panel **F**) were elicited by full-field illumination with λ = 565 nm following bleaching by intense white light (see methods).

As shown in Fig. 3A, a 1.5 ms stimulus of 14.4 log photons cm^−2^ s^−1^ intensity produced a subthreshold response. In this cell, stimulation with a 4.5 ms full-field pulse of 14.4 log photons cm^−2^ s^−1^ elicited an action potential with a latency of 9.5 ms while melanopsin-driven activity at similar or higher intensity levels is reported to have latency in the order of 1.2 s ^23^, thus ruling out the possibility of simply recording melanopsin-driven photodetection in an intrinsically photosensitive retinal ganglion cell (ipRGC). Increasing stimulus duration increased the magnitude of the depolarizing response and elicited action potentials (Fig. 3A). Prolonging stimulus duration up to 10 s resulted in a long-lasting depolarizing response, but the number of action potentials decayed with time (Fig. 3A, top trace). The number of action potentials elicited as a function of pulse duration revealing a dependence of response strength upon stimulus duration (Fig. 3B). We explored the intensity-response relationship induced by a 1 ms flash by quantifying the magnitude of the light-induced depolarization rather than number of action potentials as 1 ms pulses typically elicited only one or maximum two spikes irrespective of stimulus intensity. Figure 3C shows a linear relationship between light intensity and membrane depolarization (correlation coefficient R^2^ = 0.9359, n = 6 cells). Furthermore, the subthreshold component of optogenetically elicited light responses faithfully followed sinusoidal modulation of intensity (14.4 log photons cm^−2^ s^−1^) at frequencies between 1 and 30 Hz (Fig. 3D). Recordings from RGCs of dystrophic animals transduced with bReaChES demonstrated response patterns very similar to those of WT animals (SI Appendix, Fig. S2). Subthreshold oscillations in membrane potential closely followed the stimulus time course for frequencies between 1 and 10 Hz, with spikes present primarily in the depolarizing phase of the response, whilst a frequency of 30 Hz led to sustained depolarization, in which each cycle elicited at least one spike. Increases in stimulus frequency led to reductions in latency to first spike (Fig. 3E). To test whether or not optogenetic stimulation could precisely control spike timing and fidelity at high frequencies, we stimulated bReaChES-expressing RGCs of dystrophic retinae at 50 Hz with 1 ms flashes. Four out of 8 cells tested at this frequency could reliably follow stimulation at this frequency with spikes locked to the stimulus (Fig. 3F). To further examine the range of spiking frequencies achieved by sinusoidal stimulation, we quantified the instantaneous firing rate for a range of frequencies between 1 and 30 Hz. Instantaneous firing rate measured during the first cycle for each frequency increased linearly with stimulation frequency (Fig. 3G, r^2^ = 0.994, n = 4 cells) but when measured over the course of 1 s, it remained steady up to stimulation with 10 Hz but decayed at 30 Hz stimulation.

Another key feature of optogenetically induced responses was its efficiency to elicit short latency spikes. Short flashes (15.3 ± 0.1 log photons cm^−2^ s^−1^) elicited action potentials whereas current injections (150 pA) of the same 2 ms duration only produced subthreshold depolarizations (Fig. 3H, bottom traces). Increasing stimulus duration revealed stronger responses for light stimulation than for current injections (Fig. 3H, middle and top traces). The mean latency of bReaChES-induced responses was shorter than that of electrically-evoked responses (3.3 ± 08 vs 7.51 ± 2.6 ms, n = 5 cells) but this difference was not statistically significant.

Topological response maps were built by stimulating with an 8 × 6 grid pattern of squares (27.5 µm × 27.5 µm) centered around the soma. This approach confirmed that bReaChES-mediated RGC responses could be elicited by locally stimulating small patches of retina over and around the soma using a short duration, high-intensity flash (1 ms, 16.9 log photons cm^−2^ s^−1^) (SI Appendix, Fig. S3A). Flash delivery over the soma produced strong responses, quantified as number of spikes. Stimulation around the soma produced weaker responses whose strength in general decayed with distance to the soma. Similar maps for spike responses were observed in 4 out of 8 cells tested; the remaining 4 cells showed small amplitude depolarizing responses with slow onset that did not reach the threshold to trigger action potentials. In an area of 220 × 165 centered in the soma, stimulation of 50% of the squares produced at least one spike (n = 4, cells; (SI Appendix, Fig. S3B). Interestingly, local stimulation with these small patches reliably stimulated cells at 50 Hz, producing at least one spike per cycle in 4 out of 8 cells tested (Fig. 3F). The anticipated functional consequence is superior localization/discrimination than would be possible if stimulation of the axon elicited responses.

### Expression of bReaChES in retinal ganglion cells does not restore the pupillary light response and obliterates the normal response in wild-type mice

**Figure 4:**
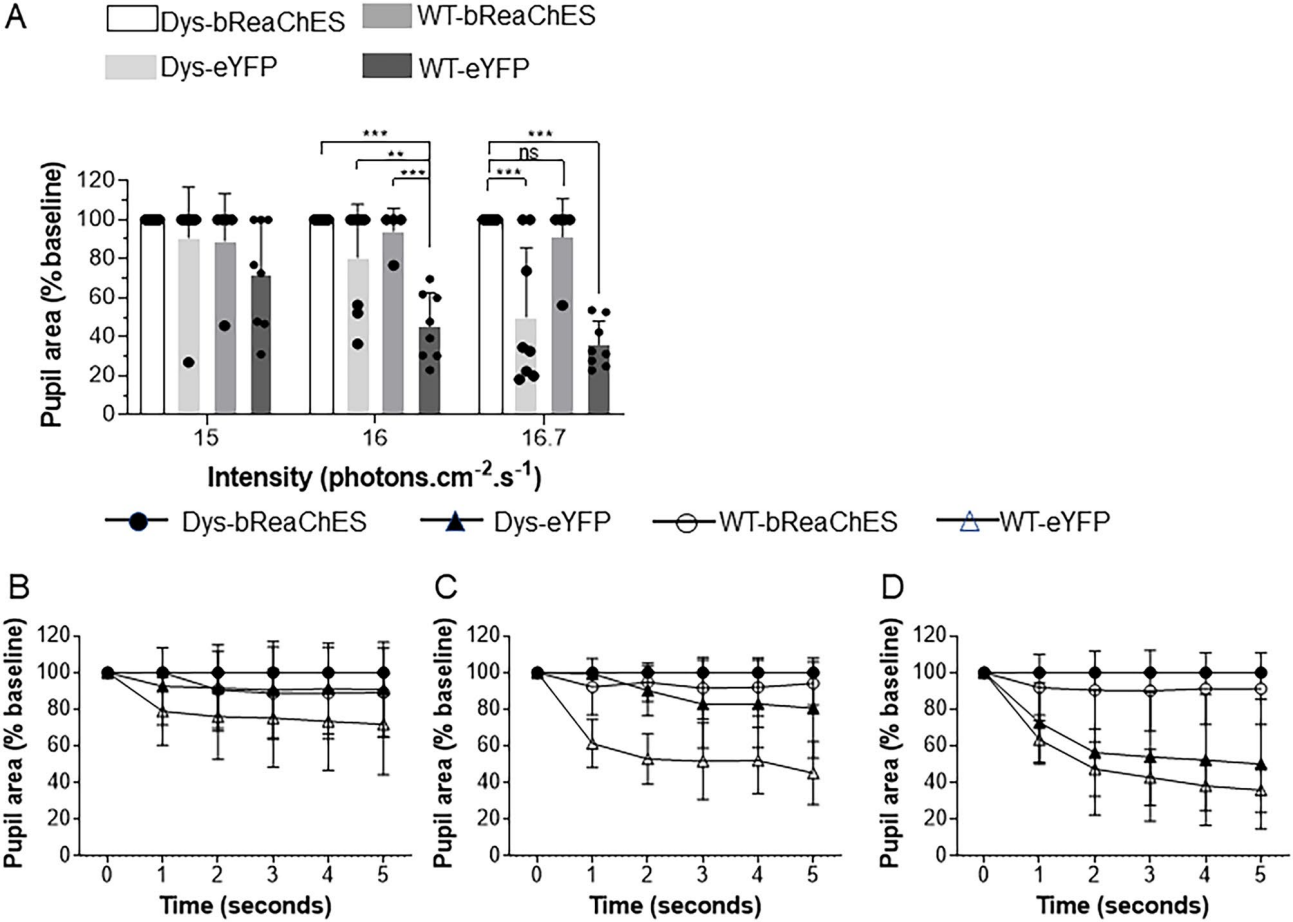
The pupil light reflex response in animals exposed to different intensities of 590 nm light stimulation 10 months after treatment. (**A**) Wild-type (WT) and dystrophic (Dys) animals treated with control vector showed constriction at all levels of stimulation, with the largest proportion of animals responded to the highest level of light stimulus following 5 s of light exposure. The percent pupil size over a 5-s period following exposure to different light intensities ranging from 1.49 (**B**), 15.9 (**C**), 16.6 (**D**) log photons cm^−2^ s^−1^. (N = 9 bReaChES-injected dys mice, N = 8 control-injected dys mice, N = 5 bReaChES-injected WT mice and N = 8 control-injected WT mice) Error bars indicate ± SD. *p < 0.05, **p < 0.001, ***p < 0.001 one-way ANOVA with one-sided Dunnett’s post hoc test.

The pupillary light response is sometimes used as evidence of restoration of light sensitivity in optogenetic gene therapy expression^14,24^, although the results are often reported to be variable^25^. In the present study, one-way ANOVA showed a statistically significant difference between groups when mouse pupillary response was measured after 5 s of exposure to light at 15.9 log photons cm^−2^ s^−1^ (*F* (3, 25) = 15.13, *p* < 0.001) and 16.6 log photons cm^−2^ s^−1^ (*F* (3, 26) = 17.294, *p* < 0.001) (Fig. 4). A subsequent Dunnett post-hoc test revealed that dystrophic and WT mice receiving control vector – but not WT mice receiving the optogenetic vector—demonstrated greater pupillary constriction than the dystrophic mice injected with optogenetic vector when exposed to light at both 15.9 and 16.9 log photons cm^−2^ s^−1^ intensities (15.9 log photons cm^−2^ s^−1^:* p* = 0.04 for dystrophic group and *p* < 0.001 for WT group, respectively; 16.6 log photons cm^−2^ s^−1^: *p* < 0.001 for both dystrophic and WT groups). Our results, therefore, demonstrate that the pupillary light reflex is abnormal in both dystrophic and WT mice treated with bReaChES vector (but not the control vector), with minimal or no pupillary responses demonstrated at light levels capable of stimulating bReaChES expressing RGCs. Collectively, these findings suggest that non-selective expression of bReaChES in the inner retina not only fails to drive ipRGC-mediated pupillary constriction in the context of outer retinal degeneration, but also interferes with the normal process of photoreceptor- and intrinsically-light sensitive retinal ganglion cell- (ipRGC) driven pupillary responses in WT animals, and ipRGC-driven responses in dystrophic animals.

### bReaChES gene therapy restores visual drive to the cortex in dystrophic mice

**Figure 5:**
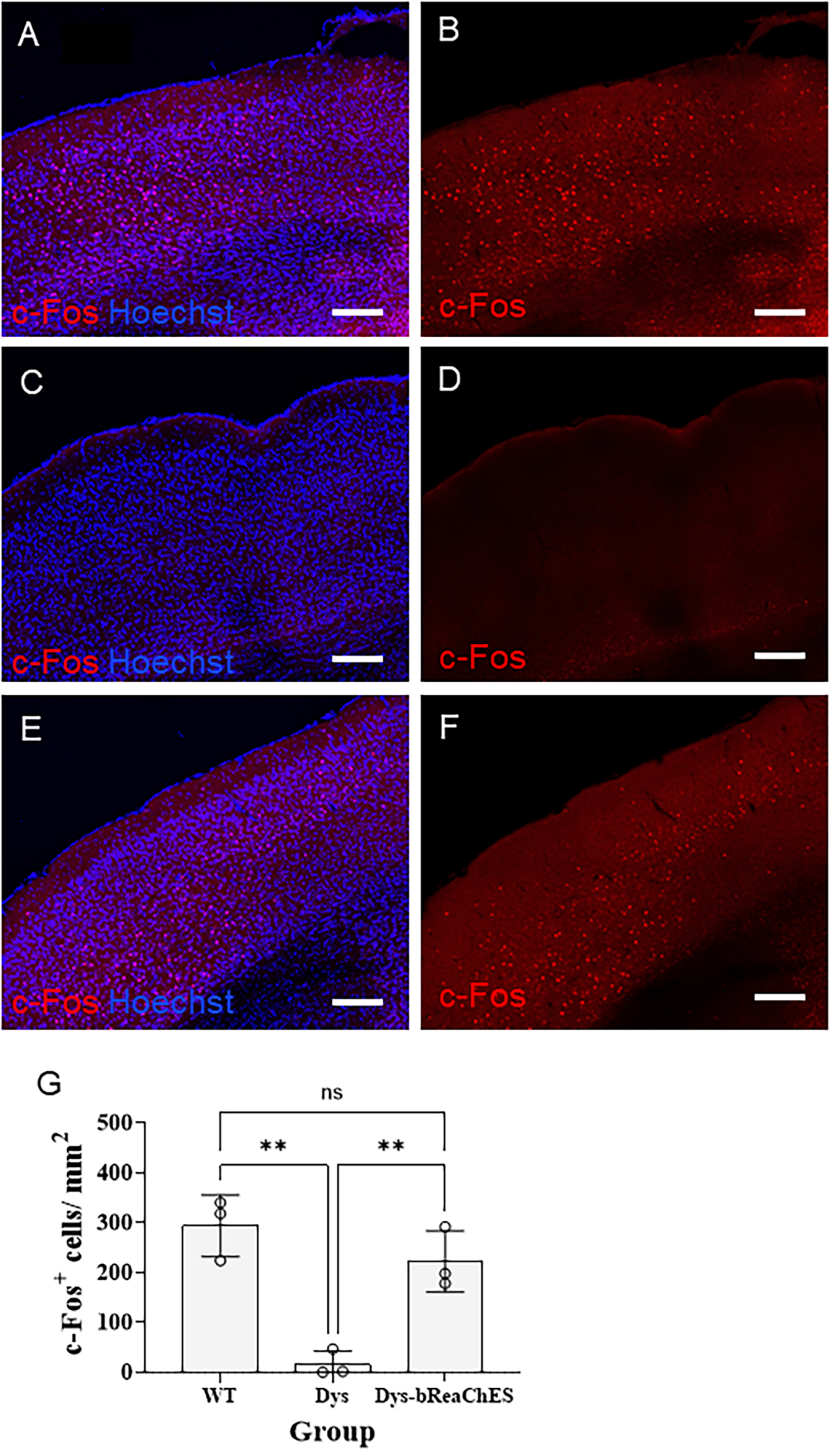
Neural activation of the visual cortex as measured by c-Fos expression following ocular light stimulation. c-Fos expression in untreated wild-type (**A**,**B**), untreated dystrophic (**C**,**D**) and bReaChES-treated dystrophic (**E**,**F**) mice. (**G**) Number of c-Fos-positive cells in the visual cortex demonstrating restoration of anatomical visual drive to the cortex in treated dystrophic mice (N = 3 mice per group). Abbreviation: WT = wild-type; Dys = dystrophic). Scale bar = 200um. Error bars indicate ± SD. *p < 0.05, one-way ANOVA with two-sided Dunnett’s test.

To further assess whether optogenetically treated dystrophic animals relay retinal inputs to the brain, the expression of the neuronal activity marker, c-Fos, was measured in the visual cortex following light stimulation. Here, a one-way ANOVA demonstrated significant group differences (F(2, 6) = 20.852, *p* = 0.008, Fig. 5), where bReaChES-treated dystrophic mice had significantly higher numbers of c-Fos-positive neurons in the visual cortex in response to light stimulation compared to untreated dystrophic mice (*p* = 0.011). On the other hand, there was no difference in c-Fos expression between bReaChES-treated dystrophic and WT mice. Furthermore, dark-adapted WT and dystrophic mice showed virtually no c-Fos activity in the visual cortex (SI Appendix, Fig. S4). These results further suggest that bReaChES expression in the RGCs of dystrophic mice restores light detection, leading to neuronal activation in the visual cortex of dystrophic mice.

### Expression of bReaChES restores behavioral responses to looming visual stimuli

**Figure 6:**
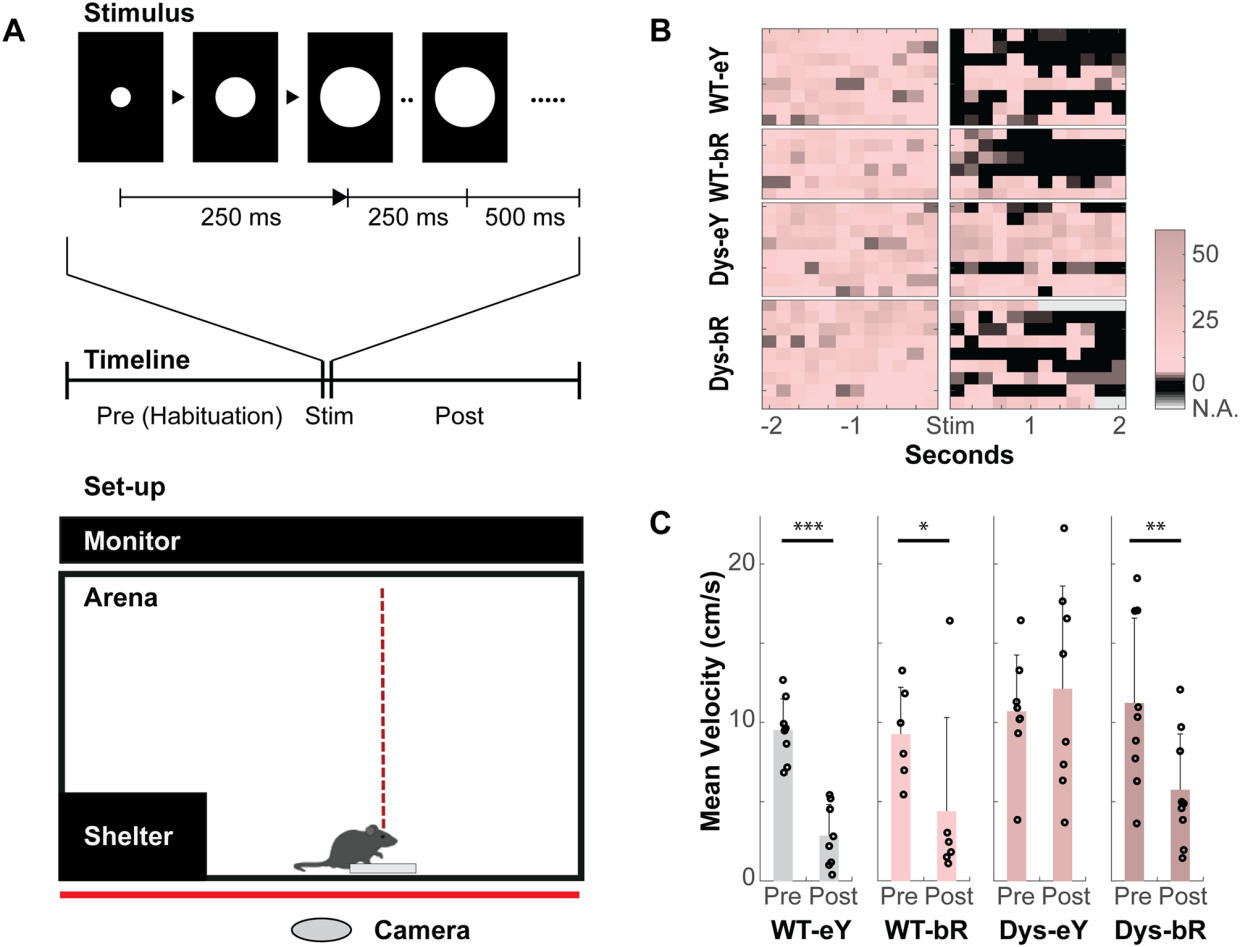
bReaChES restores visually-evoked responses in dystrophic mice. (**A**) Looming stimulus enclosure set-up. Individual mice are placed in an arena with a central dish and shelter placed in one corner. An LCD panel is positioned overhead to present stimuli. Behaviour is monitored and recorded from below using a video camera (see Methods). Top inset: schematic showing expanding stimulus presented overhead. Panels show cross-sectional images of "looming" disk. Timeline: time course of a single trial. After a period of habituation, subjects are presented with an expanding positive contrast disk (white disk (15.7 log photons cm^−2^ s^−1^) expanding at rate of 72°s^−1^ across 500 ms, with a subsequent maintenance of 500 ms (see Methods)). (**B**) Heat maps of individual mice showing instantaneous velocities 2 s before and after stimulus initiation. Note the arrest in movement (dark regions) exhibited by three of the four groups tested (WT-eYFP (WT-eY), WT-bReaChES (WT-bR), and Dys-bReaChES (Dys-bR)). (**C**) Mean velocities of movement 2 s before (pre) and 2 s after (post) stimulus onset. Dys-eYFP (Dys-eY) mice exhibited no detectable differences in motion. In contrast, Dys-bR subjects showed recovery of visually-evoked arrest. (N = 9 bReaChES-injected dys mice, N = 8 control-injected dys mice, N = 5 bReaChES-injected WT mice and N = 8 control-injected WT mice). Error bars indicate ± SD. *p < 0.05; **p < 0.01; ***p < 0.001, one-way ANOVA with Bonferroni post hoc test.

We next asked whether or not the expression of bReaChES drives the restoration of visually-guided behavioral responses in dystrophic animals and if it interferes with normal visual behavior in WT animals. When presented with overhead "looming stimuli" (Fig. 6A), mice exhibit stereotypical defensive behaviors^26,27^. To maximize the likelihood of a behavioral response mediated by bReaChES photoreception, both WT and dystrophic mice were assessed on a high contrast version of this test [i.e., a white looming disk (15.8 log photons cm^−2^ s^−1^) presented on a black background (see Methods). The unexpected presentation of a salient, visual stimulus in a dark open field overhead has been shown to drive a so-called "arrest and assess" response in rodents^28^.

Heatmaps depicting instantaneous velocities 2 s immediately before and after stimulus initiation were generated to show visually-evoked movement changes in tested mice (Fig. 6B). WT mice treated with either control or bReaChES vector exhibited a substantial decrease in movement soon after the stimulus was launched, suggesting an “arrest” response. This was similarly observed in bReaChES-treated dystrophic animals, but not in control-treated equivalents. Quantitative assessment confirmed these observations (Fig. 6C). Omnibus testing on mean velocities via a mixed model ANOVA (genotype and treatment as between-subject, and measurements 2 s before and after stimulus presentation as the within-subject factor) revealed a significant interactive effect (genotype*treatment*pre-post: F(1, 27) = 6.638, *p* = 0.016, partial h^2^ = 0.197). Bonferroni-corrected pairwise comparisons revealed that the only significant difference between genotypes was in mean velocities following stimulus onset for dystrophic and WT mice treated with the control vector (12.1 + 1.67 cm/s vs. 2.8 + 1.67 cm/s; mean ± SEM, *p* = 0.001). When considering treatments, significant differences were only observed between dystrophic mice treated with control vector and those treated with the bReaChES vector (12.1 + 1.67 cm/s vs. 5.7 + 1.57 cm/s; *p* = 0.01), with control vector treated dystrophic mice exhibiting significantly greater mean velocities following stimulus initiation. When compared across pre- and post-stimulus epochs, control- and bReaChES-treated WT mice and bReaChES-treated dystrophic mice exhibited a significant post- (compared to pre-) stimulus decrease, consistent with movement arrest upon stimulus initiation (WT-eYFP: pre (9.5 + 1.35 cm/s) > post (2.8 + 1.67;cm/s), *p* < 0.001; WT-bReaChES: pre (9.2 + 1.56 cm/s) > post (4.4 + 1.93 cm/s), *p* = 0.016; DTA- bReaChES: pre (11.2 + 1.27 cm/s) > post (5.7 + 1.57 cm/s), *p* = 0.001). On the other hand, the control-treated dystrophic mice showed no difference in mean velocities pre- and post-stimulus presentation. Together, these data indicate that the dystrophic mouse cohort treated with the bReaChES vector are exhibiting visually-evoked defensive behaviors similar to WT groups.

### Treatment AAV2 vectors drive the expression of bReaChES in the human retina

**Figure 7:**
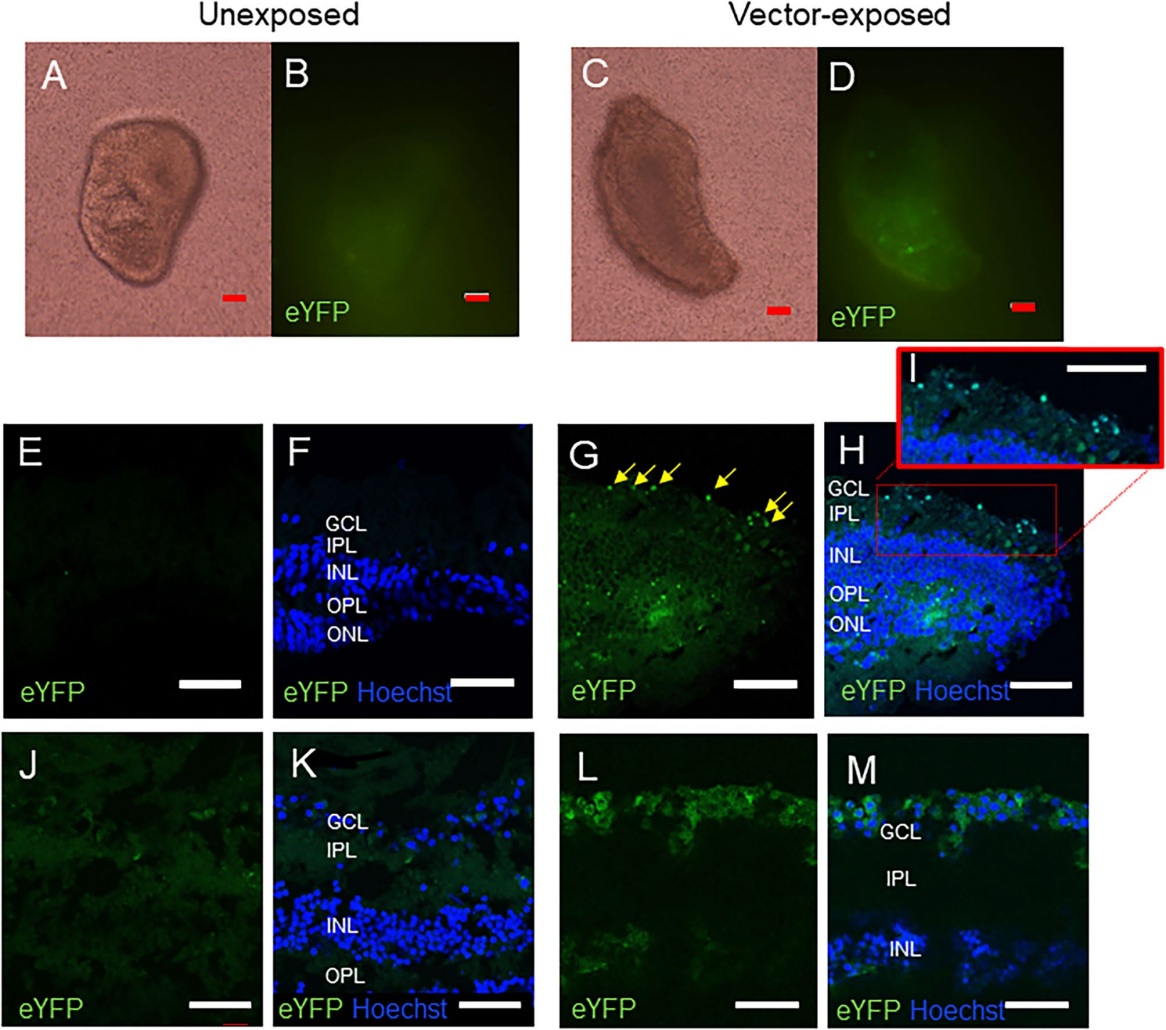
Exposure of AAV2-CaMKIIα-bReaChes-eYFP to human surgical and cadaveric retinal explants for 12 days in culture. Left column—unexposed control tissue, Right column—tissue 12 days following exposure to the treatment vector. Retinal explants viewed under brightfield (**A** and **C**) and green fluorescent channel (**B** and **D**). (**E**–**H**) Sections of human surgical retinal explants stained with Hoechst showed bReaChES-eYFP expression in vector-exposed retina (**G** and **H**). Yellow arrows indicate ganglion cells with eYFP fluorescence indicating bReaChES expression. (**H**) Colocalization of Hoechst staining and eYFP fluorescence in the GCL. Red square shows bReaChES expressing retinal ganglion cells at magnified view in inset (**I**). (**J**–**M**) Sections of human cadaveric retinal explants stained with Hoechst showed bReaChES-eYFP expression in vector-exposed retina (**L** and **M**). (**M**) Colocalization of Hoechst staining and eYFP fluorescence in the GCL. (Abbreviations: GCL = ganglion cell layer; IPL = inner plexiform layer; INL = inner nuclear layer; OPL = outer plexiform layer; ONL = outer nuclear layer). Red scale bar = 100um; White scale bar = 50um.

The ultimate aim in developing optogenetic treatment vectors is to restore vision in patients suffering from previously untreatable IRDs and atrophic macular degeneration. We, therefore, sought to determine if our bReaChES treatment vector could transduce human retina. Accordingly, we exposed cultured, living, human donor surgical explants, harvested during emergency surgery for retinal detachment repair^29^, to our treatment vector. At 12 days following exposure of excised surgical retinal explants to our treatment vector delivered in tissue culture, we were able to observe robust expression of bReaChES in surviving RGCs (Fig. 7). These results suggest that our treatment vector is capable of transducing human retina in vitro.

## Discussion

We aimed to deliver a novel type I opsin, bReaChES^18^, to the inner retina of mice with a form of severe, early-onset retinal degeneration. Further, we sought to explore whether restoration of vision would be possible via such an approach. bReaChES offers distinct advantages over short-wavelength channelrhodopsins, the first type I opsins used in optogenetic vision restoration. These include a peak sensitivity at longer visible wavelengths (peak at around 570–590 nm), making it well-matched to the spectral transmission window of the human (and, in general, the mammalian) eye and to the spectral power distribution of environmental light sources^8^. bReaChES also has high channel conductance and rapid temporal responses, which helps to confer improved light sensitivity with faster kinetics than early Type I opsins. Additionally, bReaChES possesses the inherent advantages shared by type I opsins when compared to type II opsins, including complete independence from cell-mediated processes to revert to its light-sensitive state following light activation^8^.

Fundus fluorescent photography and fluorescent microscopic imaging both demonstrated that an intravitreally injected *AAV2-CamkIIα-bReachES-TS-eYFP* viral vector resulted in strong inner retinal expression in dystrophic animals. Indeed, fundus fluorescent photography confirmed strong expression by 6 weeks post-treatment, which was sustained at 28 weeks. Concurrently, clinical fundus imaging also suggested that the treatment vector was not associated with significant intraocular inflammation and optical coherence tomography confirmed that it did not induce retinal structural changes. Fluorescent microscopic imaging suggests the preferential membrane expression of bReaChES within the neurons of the inner retina, which was sustained to 12 months post-treatment. The latter is to be anticipated given the choice of vector (AAV2), mode of drug delivery (intravitreal injection) and the promoter selected (*CamkIIα*). It is of note that the non-selective expression in inner retinal neurons conferred by this approach is much akin to the signal-agnostic stimulation of the inner retina via epiretinal electronic retinal prostheses, which have been demonstrated in clinical trials to afford functional vision restoration^7^.

Our results indicate that viral expression of bReaChes in RGCs of DTA mice does not seem to alter basic intrinsic membrane properties. Our data further suggest that the expression of bReaChES in RGCs confers novel light sensitivity to these cells. Importantly, treated cells can follow flicker of up to 50 Hz (the maximum temporal modulation of the test stimulus employed in our testing) when stimulated with 1 ms square pulses. This value is close to the temporal resolution of bReaChES predicted by modelling^30^ and approaches the temporal limit of human photopic vision, thereby suggesting that bReaChES' temporal response characteristics would be well-matched to the human visual system^31^. This is in contrast to some Type II opsin approaches, and in particular to the ectopic expression of melanopsin, which confers poor temporal resolution^14^. Optogenetically-elicited responses showed short latencies (~ 2.5 ms) and were faster than electrically-evoked responses, most likely due to the sudden reduction in membrane resistance due to the instantaneous opening of the cationic pore in the bReaChES molecule. This drop in membrane resistance in turn leads to a reduction in membrane time constant, thus leading to faster depolarization and reduction in latency to first action potential. bReaChES’ fast activation, together with its rapid off kinetics and its ability to follow high stimulation frequencies, constitute ideal properties for a candidate molecule to restore vision. Topological response maps of individual RGCs expressing bReaChES confirm responses over retinal areas ranging from 30–150 µm, fall within the range of receptive field sizes of RGCs in WT animals^32^. The rapid temporal responses of bReaChES-mediated vision restoration do not come at the expense of decreased sensitivity. The threshold for action potential generation by light activation of treated RGCs with very brief (1 ms) flashes was on average 15.3 log photons cm^−2^ s^−1^, and for longer stimuli was 13.6 log photons cm^−2^ s^−1^, which corresponds to bright indoor lighting conditions, and which is superior to the reported sensitivity of its parent molecule, ReaChR (albeit with superior temporal responsiveness)^11,12^. Furthermore, it may similarly offer superior sensitivity to the most sensitive previously described red-shifted type I opsins when expressed in second- or third-order neurons^8^, including ChrimsonR (which is currently the subject of a Phase I/II trial that has recently demonstrated proof-of-concept of the optogenetic approach in one research subject^9^). In this study we aim to characterize responses elicited by short pulse duration that would allow us to drive action potential firing at high frequencies. Responses to lower light intensities (see Fig. 3A), however, were observed for longer pulse durations. It is important to note that the spectral absorption profile of bReaChES negates one of the chief drawbacks of early type I opsin approaches (which required high illumination levels at short wavelengths): the potential for phototoxicity. Nevertheless, translation of bReaChES optogenetic gene therapy may require stimulus goggles to optimize the gain and temporal characteristics of the incoming light signal (as has been reported in the phase I/II trial of ChrimsonR optogenetic gene therapy^9^). Interestingly, instantaneous firing rates as high as 154 ± 18 Hz were observed for short periods during stimulation with either sinusoidal waves or square pulses, suggesting that modulation of pulse duration and amplitude by image encoding could be used to provide fine control of signal transmission from the retina. Optogenetic stimulation proved to elicit responses with shorter latency than electrical stimulation and achieved higher firing frequencies. However, with long stimulus durations, we observed a decay in the frequency of action potentials. This suggests that depolarization block may be an issue for constant/long duration stimuli (> 10 s), a shortcoming which could be addressed through stimulus goggles.

bReaChES expression in the inner retina failed to restore a normal pupillary light response in dystrophic animals and abolished ipRGC-mediated pupillary responses. Furthermore, we also found that its expression in WT animals abolishes the normal pupillary light response under conditions where bReaChES is anticipated to be activated by light. Our finding that non-selective optogenetic gene therapy abolishes the normal pupillary light response in WT animals is novel. What might explain these findings? Clearly non-selective expression in all subtypes of RGCs and surviving second-order neurons fails to drive a normal pupillary light response. Failure to produce a pupillary light response has been observed following optogenetic gene therapy that nevertheless restores the optokinetic reflex in triple knockout (Gnat^−/−^, Cnga3^−/−^, Opn4^−/−^) mice^24^, suggesting that restoration of vision by optogenetic approaches is not contingent upon/does not correlate to a restored PLR. However, in our animals it is clear that optogenetic photoreception inhibits the PLR. One possibility is that we are observing depolarization block of cells driving the PLR, though this hypothesis is not supported by electrophysiological recordings, which demonstrate ongoing firing of RGCs for longer duration stimuli similar to those used to elicit the pupillary light response. An alternative possibility is that this observation represents non-selective activation of different classes of RGCs and second-order neurons which are indiscriminately transduced by vectors. This could include optogenetic activation of cells that normally suppress intrinsically photosensitive retinal ganglion cells (ipRGCs), which account for the majority of cells projecting to the olivary pretectal nucleus^33^ and which drive the PLR in wild-type and dystrophic mice. For example, it is known that ipRGCs receive colossal inhibitory inputs from inhibitory amacrine cells^34,35^ and psychophysical testing in humans suggests that the S-cone pathway may inhibit ipRGCs^36^. It is furthermore possible that inhibition may have occurred, at least partially, through the activation of OFF-retinal ganglion cells which project to the olivary pretectal nucleus^33^. Although an alternative hypothesis is that exposure to the treatment vector, or the surgical procedure itself results in degeneration of treated WT retinae, our data do not support this. First, we observed no evidence of degeneration in WT animals that had undergone intravitreal injection with *AAV2-CamkIIα-bReachES-TS-eYFP*. Second, our behavioral findings suggest no difference between treatment and control vector treated WT mice. Finally, the surgical procedure itself cannot account for our pupillometry findings, as the pupillary light reflex could be obtained from WT animals treated with the control vector. Given these findings, future studies of non-selective optogenetic therapies should similarly explore the effects of treatment on pupil responses in WT animals, and on other aspects of ipRGC-mediated vision that were not assessed in the current study, such as circadian entrainment. It will also be noted that suppression of the pupillary light response may be advantageous in translation by maximizing retinal illuminance following treatment. Sahel and colleagues have developed a similar argument for using long-wavelength retinal stimulation following ChrimsonR gene therapy^9^, although it will be noted that such selective stimulation is not required to confer this benefit following *AAV2-CamkIIα-bReachES-TS-eYFP* gene therapy.

Functional restoration of vision requires an optogenetic-mediated visual signal from the retina to be reconstituted by downstream central targets. Our data indicate that visually stimulating bReaChES expressing dystrophic mice with light at an intensity well below the safety threshold (based on the recommended International Commission on Non-Ionising Radiation Protection exposure limits^37^) drove induction of immunoreactivity for the immediate early gene c-Fos in the visual cortex at 6 months post-treatment. Although we cannot confirm cortical activation by this measure alone, our observation that c-Fos expression in these treated subjects was comparable to that seen in WT animals—and significantly higher than for non-treated dystrophic mice—suggests that light is activating this pathway. Together with our behavioural observations (see below), these findings indicate that bReaChES optogenetic gene therapy restores light-evoked activation of visual circuits. Importantly, this is achieved at light levels well within the accepted safety threshold for human retina.

When exposed to overhead looming stimuli presented at light levels consistent with retinal illumination levels of 15.7 log photons cm^−2^ s^−1^, bReaChES-expressing dystrophic animals exhibited visually-guided behavioral responses. Notably, a decrease in movement was correlated with the appearance of the stimulus. This indicates that the bReaChES-expressing dystrophic subjects were aware of the stimulus. The “arrest behavior” observed was not significantly different to WT animals which received either the control or treatment vectors, suggesting recovery of visually-guided behavioural response to an ethologically relevant visual stimulus. As alluded to above, expression of bReaChES did not alter behavioral responses in bReaChES-treated WT animals compared to control-treated WT equivalents, even though these animals demonstrated disruption of the normal irradiance detecting mechanism mediated by ipRGCs and reflected in an abnormal pupillary light response. These behavioural data suggest that bReaChES does not interfere with normal visually-guided responses to temporal changes in stimulus size and mean radiance when expressed in the RGCs of WT animals. The latter finding has important implications in the context of treating individuals with localized loss of outer retinal function, for example, those with advanced loss of macular function, e.g. advanced geographic atrophy in age-related macular degeneration.

Finally, our data confirm that our treatment vector can transduce human retina in tissue culture. Although CamkIIα is up-regulated in IRD^21^, this protein is expressed in only up to 10% of normal primate RGCs^38^. Excised human surgical retinal specimens taken from patients undergoing retinal detachment surgery offer a suitable in vitro model of outer retinal degeneration because of characteristic changes in the photoreceptors and postsynaptic cleft^39,40^. Human surgical retinal explants demonstrated expression of bReaChES two weeks following exposure to the treatment vector in tissue culture. This observation is further corroborated by transduction of optogenetic vector in cadaveric human retina explants. As expression is demonstrated in human tissue in vitro, it is highly likely that expression of bRreaChES would also occur if the same viral vector were to be delivered intravitreally in vivo.

The intravitreal approach used in our study provides translational advantages over sub-retinal techniques. First, non-selective expression in inner retinal neurons targets the best-preserved cellular layers in IRD and aARMD, where there are characteristic early pathological changes present in the outer retina^8^. Furthermore, recent evidence suggests that non-selective inner retinal expression of optogenetic proteins appears to confer superior spatial discrimination to selective expression in ON-bipolar cells^25^. Second, intravitreal injection is a procedure which the majority of ophthalmologists perform. In contrast, the alternative approach of sub-retinal injection is only performed by a sub-set of surgeons who routinely undertake retinal surgery.

The type I opsin, bReaChES, has not previously been employed for vision restoration. We demonstrate that our bReaChES treatment vector is capable of long-term retinal transduction in a murine model of severe retinal degeneration, WT mice and human retinal tissue in vitro. Expression of bReaChES results in restoration of retinal sensitivity, cortical activation and behavioral responses to an ethologically relevant stimulus in dystrophic mice. This approach has numerous translation advantages: these include straightforward surgical delivery, targeting of the best-preserved cellular layer in IRD and aARMD and the potential of vision restoration with high spatial and temporal fidelity with high light sensitivity consistent with theoretical predictions of its superiority over previously employed Type I opsins^20^, making it potentially compatible with indoor lighting.

## STAR★methods: key resources table

See Table 1.

**Table 1:** Star methods resources table.

Reagent or resource	Source	Identifier
**Experimental models: Organisms/strains**		
iCre-DTA mice	This paper	NA
**Optogenetic plasmids**		
pAAV-CaMKIIa-bReaChes-TS-eYFP	Deisseroth Lab/Optogenetics innovation Lab	NA
pAAV-CaMKIIa-eYFP	Deisseroth Lab/Optogenetics innovation Lab	NA
**Antibodies**		
Lectin peanut agglutinin (PNA) conjugated with Alexa Fluor 594	Invitrogen	L-32459
Rabbit protein kinase C alpha (PKCα) polyclonal antibody	Santa Cruz Biotechnology	sc-208
c-Fos (9F6) monoclonal antibody	Cell Signalling Technology	#2250
Alexa Fluor 488 donkey anti-rabbit IgG secondary antibody	Invitrogen	A-21206
Alexa Fluor 594 donkey anti-rabbit IgG secondary antibody	Invitrogen	A-21207
**Drugs and reagents**		
Ketamine hydrochloride (Ketalar )	Parnell	NA
Medetomidine hydrochloride (Dormitor )	Pfizer Animal Health	NA
Atipamezole hydrochloride (Antisedan )	Pfizer Animal Health	NA
1% w/v tropicamide	Bausch & Lomb	NA
2.5% phenylephrine	Bausch & Lomb	NA
GenTeal moisturizing eye gel	Alcon	NA
Paraformaldehyde	Bacto Laboratories	PA00950500
Phosphate buffered saline	Medicago	09–2051-100
Normal donkey serum	Sigma	S30-100 mL
Tissue-Tek optimal cutting temperature (OCT) compound	ProSciTech	IA018
Sucrose	Univar	AJA530-500G
10% Tween 20	Bio-rad	1662404
Hoeschst stain	Life Technologies	LTS62249
Propidium iodide	Sigma-Aldrich	P4864-10ML
**Human tissue culture media**		
Neurobasal A medium	Gibco	10888022
B27 supplement	Gibco	17504044
N2 supplement	Gibco	17502048
L-glutamine	Sigma-Aldrich	G7513
Penicillin/streptomycin	Sigma-Aldrich	P4333
**Softwares**		
GraphPad Prism	GraphPad Software	Prism 8
SPSS Statistics	IBM	SPSS Statistics 26
Image J^41^	National Institute of Health, USA	1.53e
**Others**		
IR backlights	Fuloon	E8100-45-A-IR
IR camera	Canon	Model G7
PCR Thermocycler	Applied Biosystems	A37835
Micro-injector for ocular injection	Hamilton Company	Ref#7633-01
Fundus and OCT imaging system	Phoenix Technology Group	Phoenix MICRON IV
EPC8 amplifier and PatchMaster^42^	HEKA Electronik GmbH	NA
Liquid crystal display unit	LG	27MP37HQ
Cryosection station	Leica Microsystems	CM3050S
Electroretinogram system	Phoenix Technology Group	Micron Ganzfeld ERG system

## Study design and experimental model

The primary objective of the present study is to investigate the efficiency of a novel Type I opsin, bReaChES, to restore vision in a mouse model that mimics end-staged retinal dystrophy. The mouse model was produced by crossing rhodopsin-Cre mice^43^ with Rosa-DTA176 mice^44^ to induce expression of the diphtheria toxin fragment A (DTA176) gene under the control of the rhodopsin promoter in the photoreceptors, thereby ablating the photoreceptors of transgenic mice. By weaning age (~ 3–4 weeks), the transgenic dystrophic mice have lost their outer nuclear layer (SI Appendix, Fig. S5), and no demonstrable response is evident on electroretinography (SI Appendix, Fig. S6). Throughout the experimental period, age- and sex-matched WT littermates were group-housed (2–6 mice per cage) with dystrophic mice in the same temperature-controlled environment under a 12-h light/dark cycle with ad libitum access to water and food. Both WT and dystrophic mice were randomly assigned to receive bilateral intravitreal injection of either treatment construct (*CamkIIα-bReaChES-TS-eYFP*) or control construct that lacked the sequence for bReaChES (*CamkIIα-eYFP*). A sustained, long-term (up to 12 months) retinal expression of treatment and control viral constructs were established by fundus photography and confirmed by histochemistry prior to functional assessments using patch-clamp recordings, pupillary light response test, behavioural assay and c-Fos cortical expression. Expression of the treatment construct was finally confirmed in human surgical and cadaveric retinal explants: these latter experiments were approved by the South Eastern Sydney Local Health District Human Research Ethics Committee (HREC/17/POWH/537) and adhered to the tenets of the declaration of Helsinki. All animal experiments and procedures were approved by the University of Sydney Animal Ethics Committee and adhered to the NSW Animal Research Act (1985—Animal Research Regulation 2010) and the 2004 NHMRC 'Australian code of practice for the care and use of animals for scientific purposes'. Investigators performing assessments on animals were masked as to the genotype and treatment group of study animals. All animal experiments and analyses were conducted in accordance with the ARRIVE guidelines (https://arriveguidelines.org; see star methods key resources summarized in Table 1).

## Materials and methods

All methods were carried out in accordance to relevant guidelines and regulations.

### Viral vector

The expression of the type I opsin, bReaChES, was driven by a calcium calmodulin IIα (*CamkIIα*) promoter and its expression enhanced by a woodchuck hepatitis virus post-translational regulatory element (WPRE)^45^. A downstream reporter yellow fluorescent protein (*eYFP*) gene was included to confirm transduction in vivo. The *CamkIIα* promoter was selected in preference to the more commonly utilized human synapsin 1 (*hsyn1*) promoter as CamkIIα is upregulated in retinal degeneration^21^; furthermore, preliminary experiments suggested that it conferred superior expression to *hsyn1* in our animals. In addition to the treatment construct (*CamkIIα-bReaChES-TS-eYFP*), we also employed a control construct that lacked the sequence for bReaChES (*CamkIIα-eYFP*). The constructs were packaged into an adeno-associated virus 2 (AAV2) vector. Complete viral vectors were produced at the Translational Vectorology Group at Children's Medical Research Institute, Sydney, Australia or obtained from the Vector Core, University of North Carolina, USA.

### Genotyping

Transgenic animals were genotyped by PCR technique with the following primers: WS268 GTTATCAGTAAGGGAGCTGCAGTGG, WS270 AAGACCGCGAAGAGTTTGTCCTC, and WS271 GGCGGATCACAAGCAATAATAACC to amplify a WT band of 415 bp and a band of 302 bp corresponding to the *ROSA-DTA176* allele. PCR conditions are: 94 °C, 90 s, 36 cycles (94 °C 30 s; 59 °C, 45 s; 72 °C, 60 s), 72 °C, 10 min.

### Intraocular injections

Bilateral intravitreal injections of viral vectors were performed on mice at 2 months of age. Animals were anesthetised intraperitoneally (i.p.) with a mixture of ketamine (38.4 mg/kg) and medetomidine (0.48 mg/kg), and the pupils were dilated with 1% tropicamide and 2.5% phenylephrine. Under a dissecting microscope (Leica M60, Leica Microsystems Pty Ltd, NSW, AUS), 2 μL of vector (optogenetic vector rAAV2/*CamkIIα-bReachES-TS-eYFP* and control vector rAAV2/*CamkIIα-eYFP*) at concentrations of 2 × 10^12^–1 × 10^13^ viral genomes/mL were delivered intravitreally with a 32-gauge type 4 needle (Hamilton Company, Reno, NV) connected to a 5-μL Hamilton syringe model 65 (Hamilton Company, Reno, NV) as described previously (Fan J et al. Theranostics 2020). Anaesthesia was reversed by i.p. injection of atipamezole (0.96 mg/kg).

### Fundus imaging

Four to six weeks following intravitreal injection, the ocular fundus was imaged using a Phoenix MICRON IV (Phoenix Technology Group, CA, USA) fundus camera in brightfield and fluorescent modes (the latter was used to confirm, in vivo, expression of viral vectors). Before fundus imaging, mice were anaesthetized i.p. with a mixture of ketamine (38.4 mg/kg) and medetomidine (0.48 mg/kg) and their pupils were dilated with 1% tropicamide and 2.5% phenylephrine. Anesthesia was reversed by atipamezole (0.96 mg/kg).

### Single retinal ganglion cell patch-clamp recordings

Patch-clamp recordings in the whole-cell configuration were conducted on retinal flat mounts of mice 6 months post-injection using previously published protocols^46^. Briefly, mice were euthanized and their retinae were excised. The cornea, iris and vitreous humor were removed from the eye and the retina separated from the underlying choroid and sclera. Excised retinae were placed outer-retina side down in a recording chamber which was then transferred to an upright microscope (BX50 WI, Olympus Corp., Tokyo, Japan). The tissue was continuously perfused with carboxygenated AMES medium at 3–5 mL/min at 35˚C. With the aid of camera visualization, a small hole was torn in the internal limiting membrane with an empty patch pipette to gain access to the RGC layer. Whole-cell patch-clamp recordings were conducted with borosilicate glass pipettes of resistance 6–8 MΩ. High resistance seals (> 1 GΩ) were made on the cell body of RGCs. Recordings were obtained in both current and voltage-clamp conditions using an EPC8 amplifier and PatchMaster (HEKA Electronik GmbH, Lambrecht, Germany) software. Recordings were obtained with an internal solution containing (in mM) potassium gluconate: 140, HEPES acid: 10, MgCl_2_: 4.6, ATP-Na^+^: 4, GTP-Na^+^: 0.4 and EGTA: 10, pH 7.4. All ingredients purchased from Sigma-Aldrich. Input resistance was calculated according to Ohm's law (V = IR) from the change in steady-state membrane potential produced by small-amplitude, hyperpolarizing current injections (to avoid eliciting action potentials and/or activating other nonlinear conductances). Given that these measurement were made in AMES solution in the absence of any blockers, input resistance values reflect both synaptically activated and non-synaptic conductances. Spike widths were measured as the width at half-height; 6–10 of such measurements were obtained for each cell. Latency was estimated as the time between stimulus onset and the peak of the first action potential.

Responses to full-field illumination with 565 nm (Δλ = 40 nm) at different irradiance levels following bleaching by intense white light (17.8 log photons cm^−2^ s^−1^) to abolish any residual photoreceptor input were recorded to establish the threshold for optogenetic activation. Furthermore, the temporal response characteristics to sinusoidally modulated light of 565 nm presented at different temporal frequencies (1–30 Hz) was also explored and recorded. Optogenetic stimulation was also generated using a DLP system (PolyGon 400, Mightex) that allows fine spatial and temporal characterization of the responses while stimulating with a 470 nm LED (Δλ = 28 nm). Spatial sensitivity properties of individual cells were determined by systematically varying the x and y coordinates to generate topological response maps of individual RGCs expressing bReaChES. Spikes were detected off-line by calculating the smooth first and second derivative of the membrane potential signal and comparing the maxima to a threshold (usually above −35 mV). The amplitude of light-evoked postsynaptic potentials was measured after removing spikes by linear interpolation of the membrane potential signal 3 ms before each spike and ~ 5 ms after each spike. Instantaneous firing rate was calculated as the reciprocal of the interspike interval for spike trains occurring within a cycle of sinusoidal stimulation. Data analysis was carried out using custom written routines in Igor Pro (Wavemetrics, Lake Oswego, OR). Data are presented as mean ± s.e.m.

### Pupillometry

The consensual pupillary light reflex was measured during the light phase of the light–dark cycle. Mice were dark-adapted in the test room for at least 1 h immediately before testing. Briefly, an unanesthetized mouse was gently restrained to position its left eye in front of a 1-mm aperture of a ganzfeld stimulus illuminated by a 590-nm LED (Δ λ = 24 nm, Cree, North Carolina, USA) and the right eye in front of an infrared HD digital camera (Model G7, Canon, Tokyo, Japan). A portable power supply (Powertech Plus, Electus, Sydney, Australia) powered the LED to generate three different light intensities at 14.9, 15.9, 16.6 log photons cm^−2^ s^−1^ at the level of the retinal surface. Each mouse was first subjected to the dimmest light stimulus, returned to its cage and kept in darkness for approximately 20 min before re-exposure to a brighter light stimulus. The pupillary reflex was measured as the percent of baseline pupil area at 1 s intervals after the commencement of light stimulation for 5 s. Timings were regulated by a micro-controller (Raspberry Pi Foundation, Cambridge, UK).

### c-Fos expression in visual cortex

The cortical neuronal activity of mice following visual stimulation with light was assessed using c-Fos immunohistochemistry. Experiments were carried out in animals 6 months post-treated with bReaChES vector. Animals were housed in a darkened room overnight before being placed in a rectangular box (18 cm width $\times$ 20 cm length $\times$ 18 cm height), where individual mice were exposed to a yellow-filtered light generated from Schott KL200 (Schott Australia Pty. Ltd., Australia) with Omran 64,255 Tungsten halogen bulb covered with Neewer yellow filter to produce a maximum of 16.3 log photons cm^−2^ s^−1^ at the retinal level at the central ground position. After 30 min of light exposure, mice were anesthetized and transcardially perfused with PBS. Brains were removed and fixed overnight in 4% PFA at 4 °C, followed by cryoprotection in 30% sucrose for 2–3 days and snap-freezing with liquid nitrogen. Frozen brains were sectioned at 40 μm thickness through the caudorostral direction. For immunohistochemistry, each coronal brain section was blocked with 10% normal donkey serum in PBS for 1 h at room temperature. The sections were subsequently incubated with c-Fos (9F6) monoclonal antibody (1:100, #2250, Cell Signalling Technology, United States) diluted with antibody dilution buffer containing 2.5% fetal calf serum and 1% Triton X-100 in PBS for 3 days at 4 °C before being incubated with Invitrogen Alexa Fluor 594 donkey anti-rabbit IgG secondary antibody (1:1000, Thermo Fisher Scientific, NSW, AUS) for 4 h at room temperature. The sections were finally counterstained with 5 μM Hoechst 33,342 solution for 5 min at room temperature and coverslipped for confocal microscopy imaging under the same microscopic settings. To quantify c-Fos expression, three sections per brain separated 8–10 sections apart across the caudorostral cortical brain region were processed for c-Fos immunofluorescent staining. c-Fos^+^ cells were identified on a 16-bit image with threshold set from 50 to 90 (pixel value). The number of c-Fos^+^ cells was counted using the point tool of Image J 1.53e^41^ and was normalized to the area of brain (mm^2^) where quantification carried out (Raw data in Supplementary Table 2)*.*

### Behavioural assay

We employed a modified version of a previously described behavioural assay to investigate visually evoked, defensive responses in treated and control animals^17,26,27^. In this paradigm, mice are placed within a custom-built glass enclosure measuring 48 cm $\times$ 48 cm $\times$ 30 cm. The four walls of the enclosure are blacked-out to minimize extraneous light from reflections. A clear long-wavelength transmitting (red) perspex sheet is placed underneath the enclosure to permit video recording using a camera (Microsoft, WA, USA) placed underneath. The testing arena contains a shelter in one corner (12.5 cm × 10.5 cm × 7.5 cm) and a small round dish at the center measuring 6 cm in diameter, which is present during the habituation and testing phases of the experiments. A thin liquid crystal display (LCD) unit (LG IPS Monitor 27MP37HQ, Seoul, South Korea) is placed overhead at a distance of 30 cm from the enclosure floor, which corresponds to about 88° × 88° of visual angle at the corneal surface of the animal. A looming white circular stimulus (disk) is presented in positive contrast on the LCD unit: its size increasing at a rate of 72°s^−1^ (2° to 20° across 250 ms, followed by 250 ms at maximal expansion). The stimulus was repeated 15 times, with a 500 ms interstimulus interval. The retinal irradiance of the expanding disk corresponded to 15.8 log photons cm^−2^ s^−1^ at the retinal surface. WT control animals have been reported to arrest movement upon presentation of the stimulus^28^.

### Mouse tissue collection and processing

Mice were culled at 12 months after intravitreal injection of viral vectors. Whole eyes were enucleated, dissected, and immediately fixed in 4% (w/v) paraformaldehyde (Bacto Laboratories Pty Ltd, NSW, Australia) in PBS for 30 min at room temperature. Following this, opened eyecups were transferred to PBS for retinal flat-mount preparation or cryoprotected in 30% (w/v) sucrose in PBS at 4 °C overnight before embedding in Tissue-Tek optimal cutting temperature (OCT) compound (ProSciTech Pty, OLD, Australia). Embedded eyecups were frozen in liquid nitrogen and stored at −80 °C. Ocular tissue was sectioned at 10 μm using a Leica Cryostat CM3050S (Leica Microsystems Pty Ltd, Vic, Australia), mounted to Superfrost Plus slides and stored at −20 °C until further processing. Nuclear staining was performed on retinal sections by incubating with 10 μg/mL propidium iodide for 5 min at room temperature. To quantify transduction rates of RGCs, retinae were incubated with 5.5uM Hoeschst 33,342 solution for 15 min at room temperature and mounted on glass slides. Cellular bReaChES expression was then visualized using confocal imaging microscopy. Three 20 × magnified images were randomly taken from different topographical locations of each wholemounted retina. Transduced cells were counted and recorded using the point tool of Image J 1.53e^41^ (Raw data in Supplementary Table 1).

### Human retinal explants

The collection of surgical and cadaveric human retinal explants received approval from the South Eastern Sydney Local Health District Human Research Ethics Committee (HREC/17/POWH/537). To obtain surgical retinal explants, informed pre-operative consent was obtained from patients undergoing surgery for retinal detachment repair. During surgery, causative retinal tears were excised with a 23-gauge (23 g) vitrectomy cutter (Constellation Vision System, Alcon, Fort Worth, TX) set at low cut rates (500–1000 cuts/minute) using a low vacuum. Each excised fragment was aspirated with the 23 g handpiece, and several retinal fragments measuring up to ≅ 0.42mm^2^ were obtained per patient (see video). Once the retinal tear was trimmed, the vitrectomy cutter was then externalized, and its tip is introduced into the opening of a 1 mL syringe pre-primed with balanced salt solution (≅ 0.2 mL) where the surgical retinal fragments and its contents were refluxed into the syringe. Cadaveric human retinae were provided by the Australian Ocular Biobank from an anonymised donor after removal of the corneas for transplantation. A 5 mm trephine was collected on the central macular region. The surgical or cadaveric retinal tissue was transferred immediately into a tissue culture insert containing 300 μL of pre-warmed neurobasal A media supplemented with 2% B27 supplement, 1% N2 supplement, 0.8 mM L-glutamine, 100 units/mL penicillin and 100 μg/mL streptomycin. The culture insert was subsequently placed within a well of a 24-well cell culture plate containing 400 μL of supplemented neurobasal A media^47^. The tissue was exposed or unexposed to the *CamkIIα-bReachES-TS-eYFP* treatment vector before incubation. The culture plate was then placed in an incubator with 5% CO2 under controlled hypothermic conditions^29^. The media in the culture insert was topped up, and half of the media in the bottom well refreshed every alternate day for 12 days, when fluorescence from the eYFP reporter was observed under an Olympus IX71 Inverted fluorescence microscope (Olympus, NSW, AUS). At this time point, explants were collected and fixed in 4% (w/v) paraformaldehyde for 30 min and cryoprotected in 30% (w/v) sucrose for at least an hour before embedding in OCT. Embedded explants were frozen in liquid nitrogen and stored at −80 °C. Frozen explants were sectioned at 12 μm using a Leica Cryostat CM3050S (Leica Microsystems Pty Ltd, Vic, Australia), mounted to Superfrost Plus slides and stored at −20 °C. To visualize bReaChES expression at the cellular level, sections were nucleus-stained with 5 μM Hoechst 33,342 solution for 5 min at room temperature before imaging with the confocal microscope.

### Confocal microscopy

Fluorescence-stained sections were imaged on a LSM700 laser scanning confocal microscope (Carl Zeiss Microscopy, Jena, Germany) equipped with 405, 488, and 555 nm excitation lasers using Zeiss Efficient Navigation (ZEN) Black software^48^. Images were processed with ZEN Blue software^49^.

### Spectral domain-optical coherence tomographic (SD-OCT) imaging

At 3-month post-injection, the retinal structure of mice was examined using Phoenix MICRON image-guided mouse OCT2 system (Phoenix Technology Group, CA, USA). Mice were anaesthetized intraperitoneally (i.p.) with a mixture of ketamine (38.4 mg/kg) and medetomidine (0.48 mg/kg). Their pupils were dilated with 1–2 drops of 1% tropicamide and 2.5% phenylephrine. Anaesthetized mice were mounted on a maneuverable imaging platform, and the cornea was lubricated with a thin layer of GenTeal moisturizing eye gel (Alcon, Geneva, Switzerland). Three horizontal images (5.4 μm apart) per eye from nasal to temporal crossing through optic nerves were taken with B-scan mode using MICRON Reveal software^50^. Retinal thickness was measured from the internal limiting membrane to the apical face of the retinal pigment epithelium on each image using InSight software^51^. The greatest retinal thickness value was recorded for each OCT image, and three readings per eye were averaged. The mean value of averaged retinal thickness per mouse was compared between treated WT or dystrophic mice and their untreated counterparts.

### Immunohistochemistry of mouse retinal sections

Mouse retina sections prepared as described in "Mouse tissue collection and processing" at 3–4 weeks of age were stained for outer and inner segments of cones*.* The sections were washed with PBS for 10 min and subsequently blocked with 10% normal donkey serum in PBS for 1 h at room temperature. Following this, the sections were incubated overnight at 4 °C with lectin peanut agglutinin (PNA) conjugated with Alexa Fluor 594 (1:100, Invitrogen L32459, Thermo Fisher Scientific, Massachusetts, USA) and with rabbi*t* protein kinase C alpha (PKCα) polyclonal antibody (1:250, sc-208, Santa Cruz Biotechnology) diluted with antibody dilution buffer containing 1% fetal calf serum and 1% Triton X-100 in PBS overnight at 4 °C. The sections were washed thrice with 0.1% v/v Tween 20 wash buffer for 5 min each, subsequently incubated with Invitrogen Alexa Fluor 488 donkey anti-rabbit IgG secondary antibody (1:1000, Thermo Fisher Scientific, NSW, AUS) for 4 h at room temperature, followed by washing thrice with 0.1% v/v Tween 20 wash buffer for 5 min each and counterstaining with 5 μM Hoechst 33,342 solution for 5 min at room temperature. Finally, the sections were washed with PBS and finally coverslipped for confocal microscopy imaging*.*

### Electroretinogram (ERG)

Scotopic ERGs were measured using a Micron Ganzfeld ERG system (Phoenix Technology Group, CA, USA) in mice aged 3–4 weeks. Mice were dark-adapted overnight, and procedures were carried under a low red-light environment. Mice were anaesthetized and their pupils dilated using the same method as for fundus and OCT imaging. A ground electrode was placed under the skin of the tail and a reference electrode inserted under the skin of the head at the position in between the ears. Once electrodes were placed, the mouse eye was brought into contact with the nosepiece of the Ganzfeld head. A series of light stimuli at wavelengths 365 nm (UV) and 504 nm (green) were presented to each mouse’s left and right eye, respectively. Light stimuli of increasing intensity were delivered for 1 ms to each mouse eye—with varying intervals in between stimulations to restore dark adaptation—as follows: −1.7 (0.7 s interval, 10 repetitions), −1.1 (0.7 s interval, 10 repetitions), −0.5 (2 s interval, 10 repetitions), 0.7 (10 s interval, 5 repetitions), 1.3 (20 s interval, 5 repetitions), 1.9 (20 s interval, 5 repetitions), 3.1 log cd sec/m^2^ (60 s interval, 3 repetitions).

### Data analysis and statistics

All animals used in the study were pre-labelled with an ID, to which the experimenter was masked during data analysis. Statistical analysis of data was performed using GraphPad Prism version 5.01 for Windows (Graphpad Software, San Diego, CA, USA) and IBM SPSS Statistics version 26 (SPSS Inc, Somers, NY, USA). For multiple group comparisons, Bonferroni or Dunnett's test with dystrophic mice set as the "control" group (i.e. to which all other groups were compared) was carried out following a statistically significant one-way ANOVA. Two-group comparisons were performed with an unpaired *t*-test or Welch's *t*-test if the *F*-test of equality of variances found significantly different variances between the two groups. Data are reported as mean ± SD. Statistical significance was deemed to have been achieved if *p* < 0.05.

### Radiometric calculations and adjustments for bReaChES absorption

Light sources were measured using an Ocean Optics USB2000 + XR1-ES (Ocean Optics, Orlando FL) and retinal irradiance was calculated using previously described methods^52^. The efficacy of all light stimuli were corrected for bReaChES absorption by calculating the quantal catch based on published data on the spectral sensitivity of neurons expressing bReaChES^18^ and the spectral power distribution of the light sources.

## Supplementary Information

Supplementary Information.

## Data Availability

All data are available in the main text or the Supplementary Materials. All materials created in this study are available with material transfer agreements approved by the University of Sydney to any researcher for purposes of reproducing or extending the analysis.
