# Supplementary material for: Optogenetic restoration of high sensitivity vision with bReaChES, a red-shifted channelrhodopsin
Source: Sci Rep. 2022 Nov 11;12:19312. doi: 10.1038/s41598-022-23572-4 (PMC9652428; doi:10.1038/s41598-022-23572-4)
Supplement: Supplementary file 1 — Supplementary Information. [file 41598_2022_23572_MOESM1_ESM.pdf]

Supplementary Information for

Optogenetic restoration of high sensitivity vision with bReaChES, a novel red-shifted channelrhodopsin

Lay Khoon Too[1+], Weiyong Shen[1+], Dario A. Protti[3], Atomu Sawatari[3], Dylan Black[3], Catherine A. Leamey[3], Jin Huang[4], So-Ra Lee[1], Ashish Mathai[1], Leszek Lisowski[5,6], John Lin[7], Mark Gillies[1,2], Matthew P. Simunovic[1,2*]

Affiliations:

[1]Save Sight Institute, The University of Sydney; 8 Macquarie St, Sydney, NSW, 2000, Australia.
[2]Sydney Eye Hospital, 8 Macquarie St, Sydney, NSW, 2000, Australia.
[3]Neuroscience, School of Medical Sciences, Faculty of Medicine and Health, The University of Sydney; NSW, 2006, Australia.
[4]Education Innovation Theme, School of Medical Sciences, Faculty of Medicine and Health, The University of Sydney; NSW, 2006, Australia.
[5]Children's Medical Research Institute, The University of Sydney; NSW, 2006, Australia.
[6]Military Institute of Medicine, Laboratory of Molecular Oncology and Innovative Therapies; 04-141 Warsaw, Poland.
[7]School of Medicine, College of Health and Medicine, University of Tasmania; Tasmania, 7000, Australia.

[+] L.K.T. and W.S. contributed equally to this work.
[*] To whom all correspondence should be addressed: 8 Macquarie St., Sydney NSW 2000, T +612 9382 7111, F +612 9382 7114, E matthew.simunovic@sydney.edu.au

This PDF file includes:

Figure S1. Assessment of mouse retinal structure by spectral domain-optical coherence tomographic imaging.
Figure S2. Membrane potential responses to sinusoidal modulation of light at 1, 3, 5, 10 and 30 Hz (top to bottom) in a RGC of a bReaChES-expressing wild type mouse.
Figure S3. Responses of bReaChes-expressing wild-type retina to local stimulation.
Figure S4. C-Fos expression in the visual cortex of control mice that were not exposed to ocular light stimulation.
Figure S5. Immunohistohistochemical staining of protein kinase C alpha (PKCα) and peanut agglutinin (PNA) in WT (A) and dystrophic (B) mouse retina extracted at 3 weeks old.
Figure S6. Electroretinography examination of mice at 3-4 weeks old
Supplementary Table 1: Raw data of bReaChES transduction rate in whole-mounted retina.
Supplementary Table 2: Raw data of c-Fos immunohistochemistry in visual cortex.

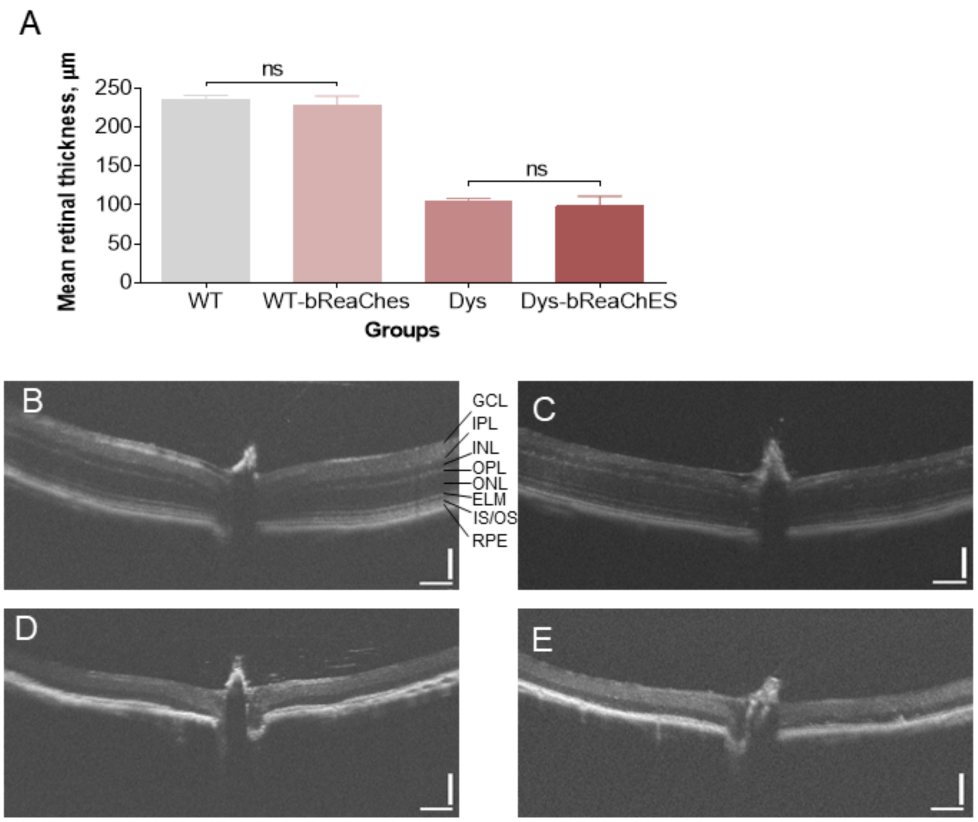

Fig. S1. Assessment of mouse retinal structure by spectral domain-optical coherence tomographic imaging. (A) Mean retinal thickness of both eyes of each mouse. Total N = 8 uninjected wild-type, 6 bReaChES-treated wild-type, 3 uninjected dystrophic, and 6 bReaChES-treated dystrophic mice. Ns = not significant, unpaired or Welch's *t*-test. (B-E) representative SD-OCT images of uninjected wild-type (B), bReaChES-treated wild-type (C), uninjected dystrophic (D), bReaChES-treated dystrophic (E) mice. Error bars indicate ± SD. Scale bar = 100um. (Abbreviations: WT = wild-type; Dys = dystrophic; GCL = ganglion cell layer; IPL = inner plexiform layer; INL = inner nuclear layer; OPL = outer plexiform layer; ONL = outer nuclear layer; ELM = external limiting membrane; IS/OS = inner segment/outer segment; RPE = retinal pigment epithelium)

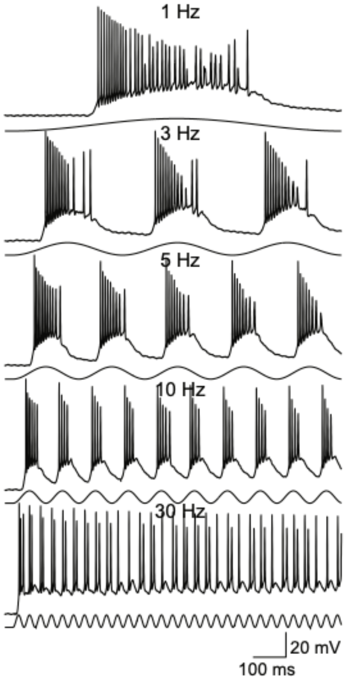

Figure S2. Membrane potential responses to sinusoidal modulation of light at 1, 3, 5, 10 and 30 Hz (top to bottom) in a RGC of a bReaChES-expressing wild type mouse. Sinusoidal stimulation resulted in reliable action potential firing for frequencies up to 30 Hz. Peak intensity of sinusoidal light stimulus = 15.6 log photons.cm^{-2}.s^{-1}.

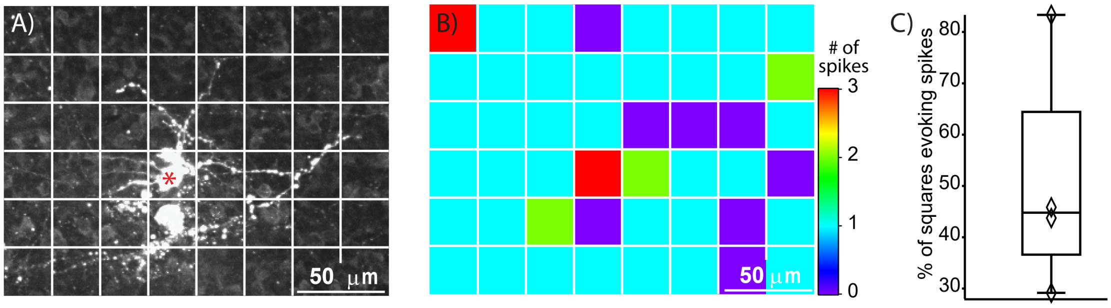

Fig. S3. Local photostimulation with small squares evokes spiking activity in bReaChes transfected RGCs. A) Confocal photomicrograph of retinal ganglion cell with overlaying 8 x 6 grid pattern used for stimulation using a random sequence of small squares (27.5 μm x 27.5 μm). A retinal ganglion cell (labeled with red asterisk) was recorded in whole-cell mode and stimulated with 1 ms square pulses of high intensity (16.9 log photons.$cm^{-2}.s^{-1}$) blue light using a digital micromirror device. B) Heatmap of spike responses. In this cell, 40 out of 48 squares delivered in an area of 220 μm x 165 μm centered around the cell elicited at least one spike. C) Percentage of squares that evoked spiking activity in cells that responded stimulation with small squares (n = 4 cells).

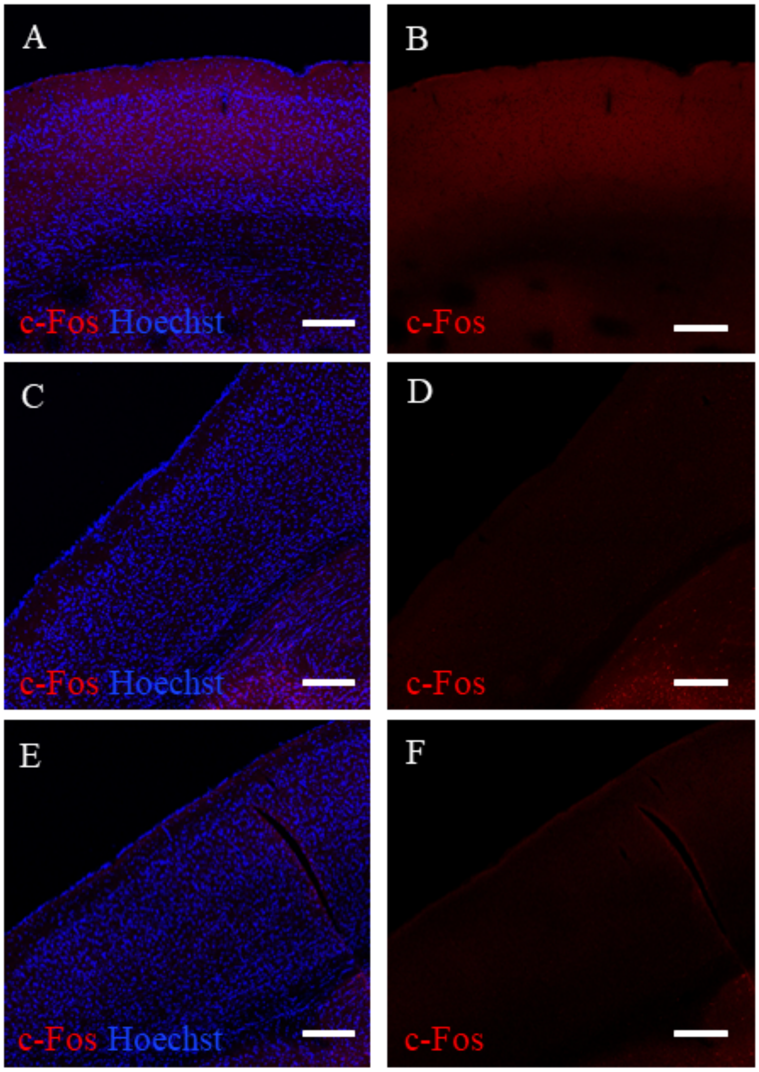

Fig. S4. C-Fos expression in the visual cortex of control mice that were not exposed to ocular light stimulation. (A-B) untreated wild-type, (C-D) dystrophic, and (E-F) bReaChES-treated dystrophic mice. Scale bar = 200um.

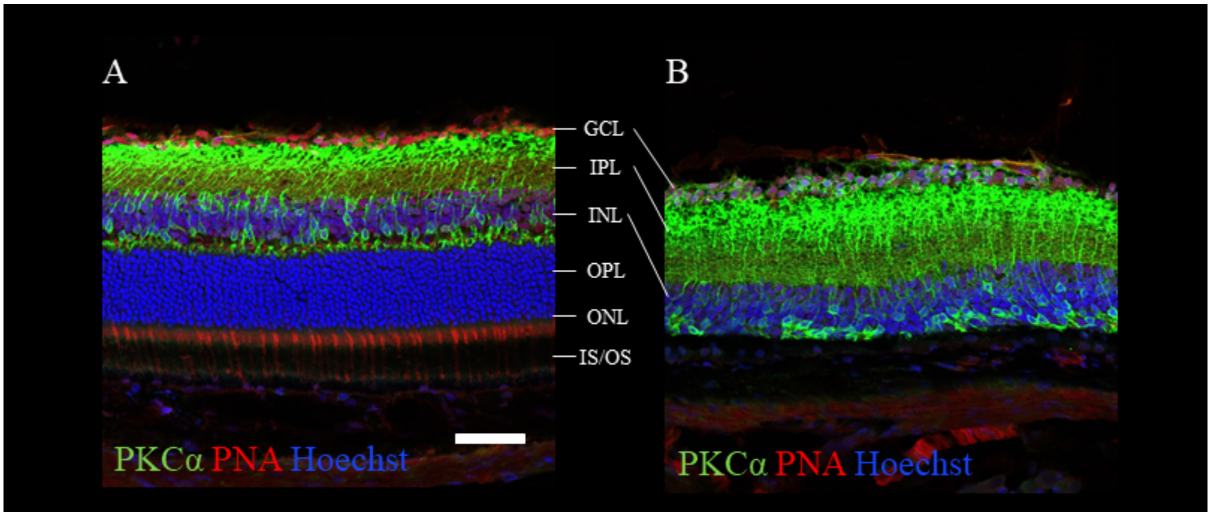

Fig. S5. Immunohistohistochemical staining of protein kinase C alpha (PKCα) and peanut agglutinin (PNA) in WT (A) and dystrophic (B) mouse retina extracted at 3 weeks old. Scale bar = 50um. (Abbreviations: GCL = ganglion cell layer; IPL = inner plexiform layer; INL = inner nuclear layer; OPL = outer plexiform layer; ONL = outer nuclear layer; IS/OS = inner segment/outer segment).

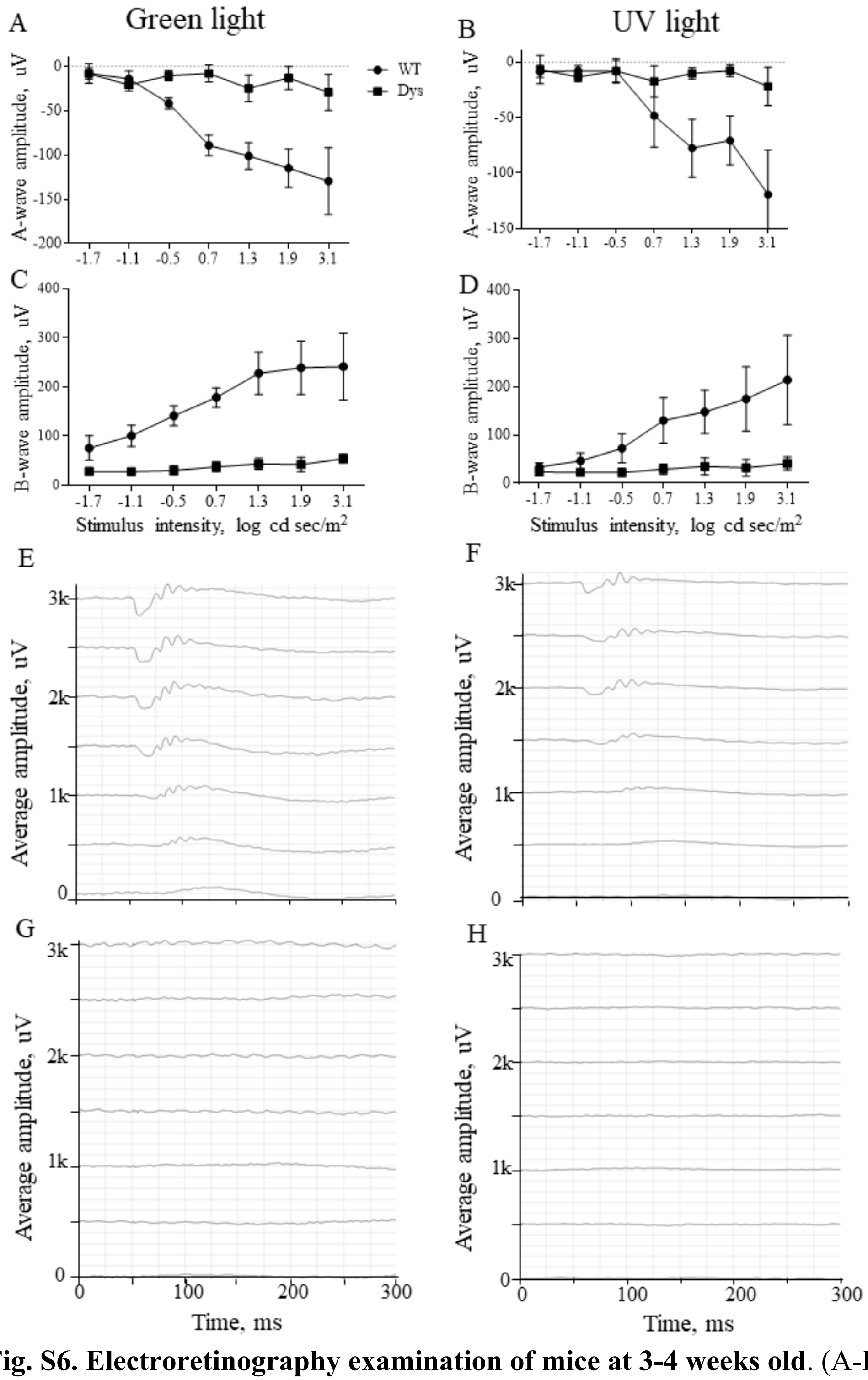

Fig. S6. Electroretinography examination of mice at 3-4 weeks old. (A-D) Amplitudes of a and b waves of wild-type (WT) and dystrophic (Dys) mice over a range of stimulation intensities. (E-H) The average amplitude of wild-type (E and F) and dystrophic mice (G and H) over 300ms following exposure to green (F and H) or UV (E and G) light stimulation. Data presented as mean ± SD.

Supplementary Table 1: Raw data of bReaChES transduction rate in whole-mounted retina

Group	Mice	bReaChES transduced RGC count	Total RGCs	bReaChES Transduction rate, %	RGCs density, cells/mm^2
Wild-type	Mouse 1	300	424	70.75	4,012
		271	418	64.83	3,955
		228	364	62.64	3,444
	Mouse 2	404	435	92.87	4,116
		408	530	76.98	5,015
		448	578	77.51	5,469
	Mouse 3	385	466	82.62	4,410
		521	592	88.01	5,602
		453	562	80.60	5,318
	Mouse 4	149	364	40.93	3,444
		204	535	38.13	5,063
		113	309	36.57	2,924
	Mouse 5	167	307	54.40	2,905
		118	326	36.20	3,085
		197	321	61.37	3,038
Dystrophic	Mouse 6	447	542	82.47	5,129
		390	507	76.92	4,798
		350	457	76.59	4,324
	Mouse 7	374	486	76.95	4,599
		309	467	66.17	4,419
		350	538	65.06	5,091
	Mouse 8	131	379	34.56	3,586
		212	429	49.42	4,060
		149	297	50.17	2,810
	Mouse 9	133	311	42.77	2,943
		72	374	19.25	3,539
		71	350	20.29	3,312

Supplementary Table 2: Raw data of c-Fos immunohistochemistry in visual cortex

Group	Mice	c-Fos$^+$ cell count	Measured area, mm^2	Cells/mm^2
Wild-type	Mouse 1	470	1.23	383
		409	1.06	387
		241	0.97	249
	Mouse 2	176	1.09	161
		217	0.88	247
		223	0.84	264
	Mouse 3	140	0.96	146
		421	0.87	484
		260	0.80	324
Dystrophic	Mouse 4	0	0.78	0
		4	0.94	4
		2	0.79	3
	Mouse 5	0	0.48	0
		0	0.92	0
		1	0.73	1
	Mouse 6	2	0.78	3
		73	0.97	75
		62	0.96	64
Dystrophic with bReaChES	Mouse 7	192	0.85	225
		456	0.71	646
		5	0.77	7
	Mouse 8	159	0.77	206
		180	0.85	211
		160	0.89	179
	Mouse 9	122	1.01	121
		201	0.91	222
		139	0.73	191
